# Variable allelic expression of imprinted genes at the *Peg13*, *Trappc9*, *Ago2* cluster in single neural cells

**DOI:** 10.3389/fcell.2022.1022422

**Published:** 2022-10-12

**Authors:** Michael Claxton, Michela Pulix, Michelle K. Y. Seah, Ralph Bernardo, Peng Zhou, Sultan Aljuraysi, Triantafillos Liloglou, Philippe Arnaud, Gavin Kelsey, Daniel M. Messerschmidt, Antonius Plagge

**Affiliations:** ^1^ Department of Molecular Physiology and Cell Signaling, Institute of Systems, Molecular and Integrative Biology, University of Liverpool, Liverpool, United Kingdom; ^2^ Institute of Molecular and Cell Biology (IMCB), Agency for Science, Technology and Research (A*STAR), Singapore, Singapore; ^3^ Department of Physiology, College of Medicine, King Saud University, Riyadh, Saudi Arabia; ^4^ Faculty of Health, Social Care and Medicine, Edge Hill University, Ormskirk, Lancashire, United Kingdom; ^5^ Université Clermont Auvergne, CNRS, Inserm, GReD, Clermont-Ferrand, France; ^6^ Epigenetics Programme, The Babraham Institute, Cambridge, United Kingdom; ^7^ Centre for Trophoblast Research, University of Cambridge, Cambridge, United Kingdom; ^8^ Wellcome-MRC Institute of Metabolic Science-Metabolic Research Laboratories, Cambridge, United Kingdom; ^9^ Institute of Cellular and Molecular Medicine, University of Copenhagen, Copenhagen, Denmark

**Keywords:** genomic imprinting, allelic expression, single-cell analysis, neurosphere, neural stem cell, *Peg13*, *Trappc9*, *Ago2*

## Abstract

Genomic imprinting is an epigenetic process through which genes are expressed in a parent-of-origin specific manner resulting in mono-allelic or strongly biased expression of one allele. For some genes, imprinted expression may be tissue-specific and reliant on CTCF-influenced enhancer-promoter interactions. The *Peg13* imprinting cluster is associated with neurodevelopmental disorders and comprises canonical imprinted genes, which are conserved between mouse and human, as well as brain-specific imprinted genes in mouse. The latter consist of *Trappc9*, *Chrac1* and *Ago2*, which have a maternal allelic expression bias of ∼75% in brain. Findings of such allelic expression biases on the tissue level raise the question of how they are reflected in individual cells and whether there is variability and mosaicism in allelic expression between individual cells of the tissue. Here we show that *Trappc9* and *Ago2* are not imprinted in hippocampus-derived neural stem cells (neurospheres), while *Peg13* retains its strong bias of paternal allele expression. Upon analysis of single neural stem cells and *in vitro* differentiated neurons, we find not uniform, but variable states of allelic expression, especially for *Trappc9* and *Ago2*. These ranged from mono-allelic paternal to equal bi-allelic to mono-allelic maternal, including biased bi-allelic transcriptional states. Even *Peg13* expression deviated from its expected paternal allele bias in a small number of cells. Although the cell populations consisted of a mosaic of cells with different allelic expression states, as a whole they reflected bulk tissue data. Furthermore, in an attempt to identify potential brain-specific regulatory elements across the *Trappc9* locus, we demonstrate tissue-specific and general silencer activities, which might contribute to the regulation of its imprinted expression bias.

## Introduction

Genomic imprinting has long been recognized as a paradigm of epigenetic regulation of a specific subset of ∼200 genes that have important roles in mammalian embryogenesis, regulation of nutrient supply and demand between mother and offspring, as well as brain development and neural functions (Ferguson-Smith, 2011; Peters, 2014; Tucci et al., 2019). Imprinted genes are defined by their parent-of-origin dependent mono-allelic or strongly biased allelic (>70%) expression. This expression bias towards a specific parental allele is a consequence of DNA methylation marks that are differentially established in either the male or female germ cells, respectively, at a defined set of CpG-rich islands (CGIs). Such germline differentially methylated regions (gDMRs) are maintained after fertilization in the somatic cells of the developing and adult offspring; they are only erased in its developing germline cells for re-setting according to its sex and transmission to the next generation (Plasschaert and Bartolomei, 2014). The germline DMRs often regulate the allelic expression of a cluster of neighboring genes and are, therefore, also called imprinting control regions (ICRs) (Ferguson-Smith, 2011; Peters, 2014; Tucci et al., 2019). In addition to DNA methylation, the mechanisms involved in the regulation of imprinted gene expression comprise histone modifications, non-coding RNAs and boundary or insulator elements that are recognized by CTCF, a methylation-sensitive DNA binding factor. CTCF binding at unmethylated sites within DMRs of imprinted genes has been shown to regulate access to tissue-specific enhancers and the formation of allelic topologically associated domains (TADs), thereby controlling the expression of neighboring genes in an allele-specific way (Bell and Felsenfeld, 2000; Lleres et al., 2019).

The imprinting status of most genes is conserved between human and mouse, although some genes do not show an allelic expression bias in one or the other species (Court et al., 2014; Tucci et al., 2019). Furthermore, while many imprinted genes have the same strong parental allele-specific expression bias in all tissues analyzed, others show tissue-specific imprinting effects, examples of which are *Gnas* in defined brain regions, endocrine glands and proximal renal tubules, *Ube3a* in neurons (but not glia and peripheral tissues), as well as *Ago2*, *Trappc9* and *Chrac1* in brain (Yamasaki et al., 2003; Weinstein et al., 2010; Mabb et al., 2011; Peters, 2014; Perez et al., 2015). The imprinting status of some genes can also be changed in specific cell types. *Dlk1* is a paternally expressed gene with important functions in adipose tissue development, metabolic regulation and neurogenesis (Peters, 2014). However, its imprinting status is lost in neural stem cells (NSCs) of the subventricular zone and hippocampal dentate gyrus with bi-allelic expression of *Dlk1* being required for postnatal and adult neurogenesis in these stem cell niches (Ferron et al., 2011; Montalban-Loro et al., 2021). The mechanism for this change in allelic expression status involves postnatal gain of methylation at the gDMR/ICR of the locus (Ferron et al., 2011).

The assessment of allelic expression biases of imprinted genes requires the generation of hybrid mice in crosses between different strains that carry a sufficient number of single nucleotide polymorphisms (SNPs) to be able to identify the parental alleles. Until recently, it was only possible to investigate the allelic expression of genes on the level of bulk primary cell culture or tissue lysates, which often contain different cell types. Although many imprinted genes show an almost exclusive parental bias of ∼90% and are considered mono-allelic in their expression, recent RNA-seq studies have revealed a number of genes with a weaker parental bias of ∼70%, which was used as a threshold for inclusion in the ‘imprinted gene’ category (Babak et al., 2015; Bonthuis et al., 2015; Crowley et al., 2015; Perez et al., 2015; Bouschet et al., 2016; Andergassen et al., 2017; Huang et al., 2017). These data raise questions about how such bulk tissue-level expression biases are reflected on a single cell level, and about the biological significance of such findings (Perez et al., 2016). Several single-cell expression scenarios can potentially lead to a parental allele-specific expression bias of ∼70% in tissues (Bonthuis et al., 2015; Perez et al., 2016), including 1) all cells show the same biased bi-allelic expression, 2) the tissue consists of a mixture of cells with mono-allelic and equal bi-allelic expression, 3) the tissue consists of an unbalanced mixture of cells with respectively mono-allelic paternal and mono-allelic maternal expression, which can be due to differential promoter usage as is the case for *Grb10* in neurons versus glial cells and peripheral tissues (Yamasaki-Ishizaki et al., 2007; Sanz et al., 2008; Garfield et al., 2011). To address this question, two types of approaches have been applied recently. Using SNP-FISH *in situ* hybridization, which employs SNP-specific oligonucleotides, Ginart et al. (2016) were able to distinguish mono-versus bi-allelic expression of *H19* and *Igf2* in fixed fibroblasts and heart tissue *via* imaging. With a different *in situ* hybridization technique, using intronic RNAscope probes for nascent RNA in nuclei, Bonthuis et al. (2015) showed a mixture of cells with mono-allelic and bi-allelic expression of specific imprinted genes in brain sections. Novel single-cell RNA-seq methods have also been applied to analyze imprinted gene expression in single cortical neurons after labeling them with fluorescent proteins and FACS sorting (Laukoter et al., 2020). Their findings indicate some degree of variability of allelic expression in individual neurons, depending on the imprinted gene analyzed. While *Meg3* and *Snrpn* showed the expected mono-allelic expression in almost all neurons, *Inpp5f* and *Impact* were mono-allelic paternally expressed in the majority of cells with smaller numbers of neurons displaying bi-allelic or even mono-allelic maternal expression (Laukoter et al., 2020). Thus, these initial studies indicate that the imprinted expression status of a gene, as determined on a tissue level, might not be reflected in all its cells.

The *Peg13-Kcnk9-Trappc9* imprinting cluster (schematically shown in Figure 6A) on mouse chromosome 15/human chromosome 8 consists of several genes with neurodevelopmental functions; mutations of these cause disorders in both species. At the center of the locus, the non-coding RNA *Peg13* is expressed from the paternal allele, starting at an unmethylated CGI promoter and DMR located within an intron of *Trappc9* (Smith et al., 2003; Ruf et al., 2007). Germline-derived methylation silences *Peg13* on the maternal allele. Complete deletion of *Peg13* on the unmethylated paternal allele is lethal in mice, while the same mutation on the maternal allele does not cause a phenotype (Keshavarz and Tautz, 2021). A milder behavioral phenotype is observed with a mutation that truncates the non-coding RNA (Keshavarz and Tautz, 2021). The second gene of the cluster, *Kcnk9*, for which imprinting is conserved between human and mouse, encodes the two-pore domain potassium channel subunit Task3 (Court et al., 2014). *Kcnk9* is expressed with a strong maternal allelic bias in adult brain (Ruf et al., 2007; Court et al., 2014; Cooper et al., 2020). Mutations of the gene cause Birk-Barel intellectual disability syndrome in humans and behavioral abnormalities in mice (Linden et al., 2007; Barel et al., 2008; Cooper et al., 2020). The three genes *Trappc9*, *Chrac1* and *Ago2* show an imprinted bias of expression from the maternal allele in mouse brain, but are not imprinted in human (Court et al., 2014; Babak et al., 2015; Bonthuis et al., 2015; Crowley et al., 2015; Perez et al., 2015; Bouschet et al., 2016; Andergassen et al., 2017; Huang et al., 2017). The mechanisms underlying the predominantly maternal expression of these three genes in mouse brain are currently unclear. *Trappc9* encodes a subunit of the intracellular trafficking protein particle II complex (TrappII), mutations of which lead to a neurodevelopmental disorder in humans and mice, which includes symptoms of postnatal microcephaly, intellectual disability and speech impairment (Ke et al., 2020; Liang et al., 2020; Wilton et al., 2020; Aslanger et al., 2022). Ago2 forms a subunit of the RNA-induced silencing complex (RISC). Heterozygous mutations in humans result in a range of neurological phenotypes while homozygous mutation in mice is embryonic lethal (Liu et al., 2004; Lessel et al., 2020). Little is known about the chromatin accessibility factor Chrac1.

In this study, we analyzed the allelic expression of these genes in bulk tissue and neurosphere lysates comparatively to single NSCs and differentiated neurons. We especially focused on *Peg13*, *Trappc9* and *Ago2* as examples of strongly and moderately biased imprinted genes. We found variability of allelic expression in individual cells, which was more pronounced for *Trappc9* and *Ago2* than for *Peg13*. All categories of expression from mono-allelic maternal to equal bi-allelic to mono-allelic paternal, as well as biased bi-allelic states, were identified for *Trappc9* and *Ago2* in single cells. For *Peg13*, a majority of cells showed the expected mono-allelic paternal or paternally biased bi-allelic expression, but a small number of cells deviated from this status, displaying equal bi-allelic or even a maternally biased expression. However, considering the whole population of single cells analyzed, we find that the imprinted gene expression status approximates the findings from bulk tissue lysates. Additionally, we determined the transcriptional start site of *Trappc9* and investigated potential transcript variants as well as regulatory regions located within the locus, which led to the identification of sequence elements with a silencing function in primary neurons and/or fibroblasts.

## Materials and methods

### Animals

Mouse strains C57BL/6J and Cast/EiJ were bred and maintained in the Babraham Institute Biological Support Unit. Ambient temperature was ∼19–21°C and relative humidity 52%. Lighting was provided on a 12 h light: 12 h dark cycle including 15 min “dawn” and “dusk” periods of subdued lighting. After weaning, mice were transferred to individually ventilated cages with 1–5 mice per cage. Mice were fed CRM (P) VP diet (Special Diet Services) *ad libitum* and received seeds (e.g., sunflower, millet) at the time of cage-cleaning as part of their environmental enrichment. Breeding and maintenance of these strains were performed under licenses issued by the Home Office (United Kingdom) in accordance with the Animals (Scientific Procedures) Act 1986 and were approved by the Animal Welfare and Ethical Review Body at the Babraham Institute. Tissues were collected from newborn or adult mice and either frozen for molecular biology or processed for cell culture. Frozen tissues from C57BL/6J and *Mus musculus molossinus* JF1 hybrid mice were kindly provided by Dr Philippe Arnaud, Université Clermont Auvergne, France.

### Neurosphere, primary neuron and fibroblast culture

Neurosphere culture was performed as described previously (Ferron et al., 2007; Chojnacki and Weiss, 2008) with slight modifications. Briefly, hippocampi were dissected from newborn mouse brain in ice-cold neurosphere growth medium (DMEM/F12 (Gibco) supplemented with 0.6% w/v glucose, 0.1% NaHCO_3_, 5 mM HEPES, 2 mM L-GIn, 100 U/ml penicillin, 0.1 mg/ml streptomycin, 1x B27 (Gibco), 10 ng/ml FGF-2 (Peprotech), 20 ng/ml EGF (Peprotech), 4 mg/ml BSA (Sigma) and then transferred into Accutase (Gibco) for dissociation into a single-cell suspension by gentle trituration. Following centrifugation at 200 g for 5 min, cells were resuspended in growth medium and plated at a density of 3,000 cells/cm^2^ in suspension cell culture dishes (Corning). Neurospheres were allowed to grow for 6–8 days with intermittent medium supplementation before passaging *via* Accutase dissociation. Cells were re-plated at a lower density of 1,500–2,000 cells/cm^2^. Neurospheres could be stored in liquid nitrogen after freezing in growth medium with 10% DMSO. For bulk or single-cell gene expression analysis, neurospheres at early passage numbers (P3–P5) were used. Neurospheres were differentiated into neurons at the point of passaging by seeding a single cell suspension on Poly-L-Lysine (Sigma) coated dishes in differentiation medium (growth medium without EGF, FGF-2 and BSA, but containing 1% FBS). For single-neuron analysis, selection against replicating glial cells was started after 2 days of culture with 2 μM Cytosine β-D-arabinofuranoside (AraC) (Sigma) as described in the next paragraph.

Primary hippocampal neurons were cultured as described (Beaudoin et al., 2012; Ioannou et al., 2019) with modifications. Hippocampi were dissected from newborn mouse brain in ice-cold dissection medium [HBSS (Sigma) supplemented with 0.1% w/v glucose, 10 mM Hepes pH 7.4, 1% Na-pyruvate] and the tissue dissociated by adding an equal volume of 2x Papain stock solution (Worthington) at 37°C for 20 min. The supernatant was removed carefully, the tissue gently washed with plating medium [MEM (Gibco) supplemented with 0.45% glucose, 10% FBS, 1% Na-pyruvate, 2 mM Glutamine, 100 U/ml penicillin, 0.1 mg/ml streptomycin] and then carefully triturated in fresh plating medium. The dissociated tissue was rinsed through a 70-μm cell strainer (Corning) and the collected cells centrifuged at 200 g for 5 min. Cells were resuspended in neuronal medium [Neurobasal medium (Gibco) supplemented with 2 mM glutamine, 100 U/ml penicillin, 0.1 mg/ml streptomycin, 1x B27 (Gibco)] and plated in Poly-L-Lysine (Sigma) coated dishes at a density of 60,000 cells/cm^2^. Medium was replaced the following day, and on day two selection against replicating non-neuronal cells was started with neuronal medium containing 2 μM AraC. Half the medium was replaced with fresh neuronal medium every other day to dilute out the AraC.

Mouse embryonic fibroblasts were prepared as described (Matise et al., 2000) and cultured from frozen stocks in Hepes-buffered DMEM (Sigma) supplemented with 10% FBS, 2 mM Glutamine, 100 U/ml penicillin, 0.1 mg/ml streptomycin.

### Cell transfections and reporter gene assays

The promoter-reporter gene plasmids were transfected into fibroblasts and into primary hippocampal neurons after 7 days of culture using Lipofectamine 2000 (Invitrogen). The firefly luciferase-based test constructs were mixed with a Renilla luciferase control plasmid (pGL4.74, Promega) at a 100:1 ratio to normalize for transfection efficiency. Cells were lysed 48 h after transfection and luciferase activities were measured using the Dual-Luciferase^®^ Reporter Assay System (Promega) on a Glomax Multi Detection System (Promega).

### RNA isolation, RT-PCR and 5′-RACE

RNA was isolated from neurospheres and tissues using TRIzol™ reagent (Invitrogen) or RNeasy Plus Mini kit (Qiagen). Samples were treated with DNAse I to remove any traces of DNA before cDNA was synthesized with ProtoScript^®^ II Reverse Transcriptase (New England Biolabs) or SuperScript III™ Reverse Transcriptase (Invitrogen) using random hexamer primers, if not otherwise stated. PCR was performed using GoTaq^®^ Hot Start Polymerase (Promega) or Q5™ High-Fidelity DNA Polymerase (New England Biolabs). For the bulk neurosphere and tissue gene expression analysis we used 1 μg of total RNA in reverse transcription reactions, which were then diluted 5-fold for endpoint PCR (initial denaturation 98°C, 30 s; 30 cycles of 98°C, 50–72°C annealing (primer dependent); 72°C, 30 s; final extension 72°C, 2 min) to obtain enough products for pyrosequencing analysis. 5′-RACE experiments were undertaken with ExactSTART™ Eukaryotic mRNA 5′&3′ RACE Kit (Epicentre/Illumina) on adult mouse brain RNA. The 5′-RACE cDNA was then amplified with a *Trappc9*-specific reverse primer (Pr_05RV; Supplementary Table S1) and a kit-supplied RACE 5′-linker primer, followed by cloning of PCR products into TOPO®-plasmids (Invitrogen) and sequencing.

### Pyrosequencing

SNPs in cDNA from tissues and neurospheres of hybrid mice, as well as genomic DNA for methylation analysis after bisulfite treatment, were sequenced using a PyroMark^®^ Q96 ID instrument (Qiagen). PCR and sequencing primers (Supplementary Table S1) were designed using PyroMark Assay Design Software 2.0. Biotinylated PCR products were immobilized on streptavidin-coated beads for cleanup and sequenced using PyroMark^®^ Gold Q96 reagents (Qiagen) following the manufacturer’s protocols.

### Single-cell isolation and allelic expression analysis

Single C57BL/6J × Cast/EiJ neurosphere cells were obtained *via* dissociation with Accutase, dilution in growth medium and either FACS sorting or manual isolation *via* capillary action under a microscope using a protocol originally developed for oocytes (Lorthongpanich et al., 2013; Cheow et al., 2015; Cheow et al., 2016). Single neurons from differentiated neurospheres were collected after 7 days of culture in differentiation medium, which included 5 days of AraC treatment. Neurons were dissociated from culture dishes using Trypsin/EDTA (Sigma), diluted in differentiation medium and single cells isolated manually *via* capillary action under a microscope. Single cells were transferred into PCR tubes containing 5 μl of lysis buffer [CellsDirect Resuspension and Lysis Buffer, 10:1 (Invitrogen)] and incubated at 75°C for 10 min (Lorthongpanich et al., 2013; Cheow et al., 2015; Cheow et al., 2016). cDNA was synthesized at 37°C by adding an equal volume of a 2x reverse transcription master mix using MultiScribe™ Reverse Transcriptase (Invitrogen) and hexamer primers. This was followed by protease treatment (Qiagen Protease) as described (Lorthongpanich et al., 2013; Cheow et al., 2015; Cheow et al., 2016) to remove chromatin-associated proteins from genomic DNA. Next, we perform single-cell restriction analysis of methylation (SCRAM), using BstUI, to digest unmethylated CpG sites of genomic DNA. This provides the additional option of analyzing DNA methylation of CGIs in the single cells (Lorthongpanich et al., 2013; Cheow et al., 2015; Cheow et al., 2016). A final Proteinase K digest was carried out before undertaking multiplex PCR pre-amplification using a pool of primers for all target genes with the following conditions: initial denaturation 95°C, 10 min; 30 cycles of 95°C, 30 s; 60°C annealing/extension, 4 min. After pre-amplification, unincorporated primers were removed from the reactions by adding exonuclease I (Lorthongpanich et al., 2013; Cheow et al., 2015; Cheow et al., 2016). Pre-amplification reactions were then diluted 10-fold and aliquots were used to amplify individual target genes *via* nested-primer qPCR using 3 μl of diluted template and PowerUp SYBR Green 2x Master mix (Invitrogen) under the following conditions: initial denaturation 95°C, 10 min; 30 cycles of 95°C, 15 s; 60°C annealing/extension 1 min; melt curve analysis 60–95°C ramp 5 s/degree. Primers for target genes are listed in Supplementary Table S1. Where applicable, these qPCR products were purified using MinElute PCR^®^ purification kit (Qiagen) and Sanger-sequenced for cDNA SNP expression analysis.

### Genomic DNA isolation, bisulfite treatment and DNA methylation analysis

Genomic DNA was isolated from tissues through Proteinase K (100 μg/ml) digest in lysis buffer (100 mM Tris, 5 mM EDTA, 200 mM NaCl, 0.2% SDS, pH 8.5) at 55°C overnight, followed by Phenol/Chloroform extraction, Ethanol precipitation and resuspension in TE buffer. Neurosphere DNA was obtained using TRIzol™ reagent (Invitrogen) in a follow-on step after initial RNA isolation *via* Phenol/Ethanol extraction, precipitation and resuspension in TE. For DNA methylation analysis of CpG sites, bisulfite conversion of unmethylated cytosines was carried out using the EZ DNA Methylation-Gold™ kit (Zymo Research) according to manufacturer instructions. The bisulfite-treated and purified DNA was then used for PCR amplification of CGI fragments, followed either by methylation analysis *via* direct pyrosequencing or cloning of PCR products into TOPO®-vectors (Invitrogen) and Sanger sequencing of individually cloned plasmid samples. Sanger sequencing results were further analyzed using the free online tool “QUantification tool for Methylation Analysis” (QUMA; http://quma.cdb.riken.jp/).

### Identification of potential regulatory elements and generation of reporter-gene vectors

Potential brain-specific gene regulatory elements were identified from histone modification and CTCF ChIP assay data, as well as DNAse I and ATAC-seq hypersensitivity data for the newborn (P0) mouse brain in comparison to peripheral tissues. These data were extracted from the databases ENCODE3 (https://www.encodeproject.org/), UCSC Genome Browser (https://genome-euro.ucsc.edu/) and ENSEMBL (https://www.ensembl.org/Mus_musculus/Info/Index). Seven candidate brain-specific regulatory elements across the *Trappc9-Peg13* locus were found; their genomic positions (mouse GRCm38/mm10 genome version) and features are listed in Supplementary Figure S6. Similarly, the *Trappc9* promoter characteristics at exon 1 were noted. To test the functionality of these regulatory elements in transfected cells, promoter-reporter gene plasmids were generated. The regulatory elements were amplified from C57BL/6J genomic DNA using Q5™ High-Fidelity DNA Polymerase (New England Biolabs), cloned into TOPO®-plasmids (Invitrogen) and sequenced for confirmation. Similarly, four *Trappc9* promoter fragments of different lengths were cloned (positions 73,061,805–73,060,204 bp, 73,061,805–73,060,975 bp, 73,061,418–73,060,204 bp and 73,061,418–73,060,975 bp in GRC38/mm10). The firefly luciferase-encoding pGL4.23 [*luc2*/minP] vector (Promega) was used to generate reporter-gene constructs. First, the endogenous minimal promoter was removed *via* HindIII and NcoI digest and then replaced with a *Trappc9* promoter fragment. The four *Trappc9* promoter plasmids were tested in a preliminary reporter gene assay for their activity. The promoter fragment 73,061,418–73,060,975 bp, which avoids an upstream dinucleotide repeat sequence stretch and ends before the exon 1 splice donor site, showed the highest activity and was used in further experiments in combination with the identified regulatory elements. The regulatory elements were cloned into the pGL vector at the BamHI site downstream of the *Luc2* reporter gene to reflect the same relative orientation to the *Trappc9* promoter as in the genome. Only the Reg-E element was cloned upstream of the *Trappc9* promoter as shown in Figure 6B.

### Statistical analysis

The data for the promoter-reporter gene assays were analyzed using GraphPad Prism v.9.3 software. Data were analyzed for outliers using the ROUT method. The datasets were then analyzed for normality using the Shapiro-Wilkinson test. For non-parametric datasets, Mann-Whitney U-tests were performed in comparisons of the Reg-element datasets to the basic *Trappc9* promoter dataset. For parametric datasets, unpaired *t*-tests were performed.

## Results

### Alternative exons and transcriptional start site of *Trappc9*

**FIGURE 1:**
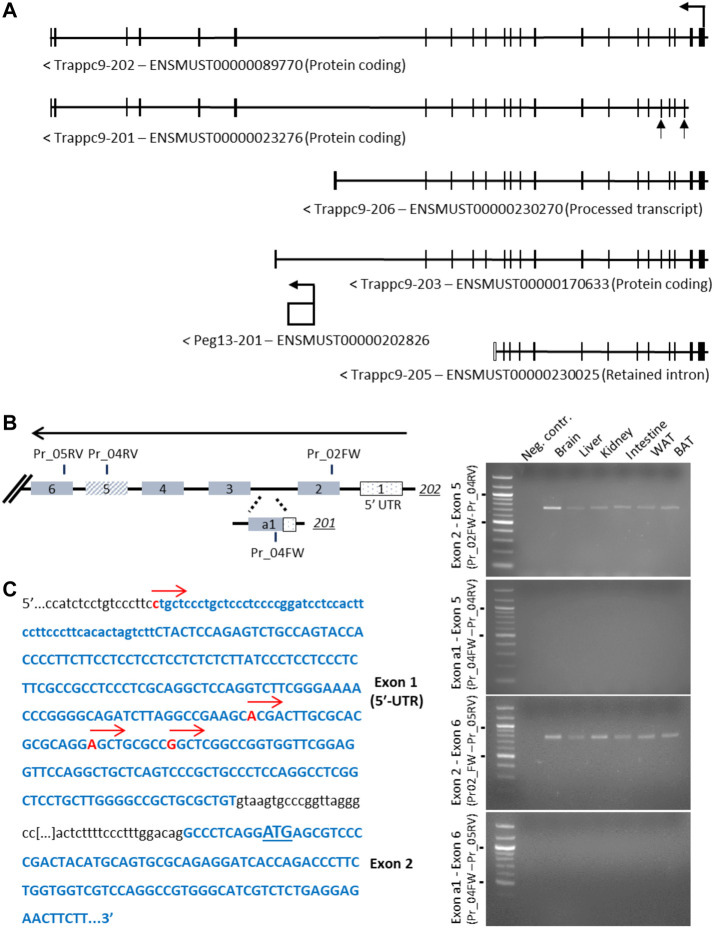
Analysis of alternative 5′-exons and transcriptional start sites of *Trappc9*. **(A)** Scheme of transcript variants *202*, *201*, *206*, *203* and *205* as indicated on ENSEMBL. The alternative exons of variant *201*, a1 and 5, are indicated by arrows. **(B)** Scheme of exons and primers used in RT-PCR (right) on total C57BL/6J RNA from multiple tissues using primer combinations specific for transcript *202* exon 2 or transcript *201* exon a1 with shared downstream primers in exons 5 and 6 (indicated in the scheme with FW and RV annotations). While expression of transcript *202* exons 2–5 and 2–6 (expected: 740 bp and 780 bp, respectively) was confirmed in multiple tissues, no expression involving transcript *201* exon a1 was detectable (expected: 318 bp and 358 bp, respectively). Size markers of 500 and 1,000 bp are indicated. **(C)** Sequencing of 5′-RACE RCR products using a downstream primer in exon 6 confirmed multiple transcription start sites in *202* exon 1 (indicated by red arrows), while no start sites in the region of *201* exon a1 could be detected. The first codon is underlined in exon 2.

The core imprinted gene of this cluster on mouse chromosome 15, *Peg13*, is located within intron 17 of *Trappc9* and is transcribed into a non-coding RNA (Smith et al., 2003; Wang et al., 2008). For *Trappc9* itself, several alternatively spliced and truncated transcript variants have been described and annotated on ENSEMBL, including alternative first exons (Figure 1A). Furthermore, while some transcript variants were found to be predominantly expressed from the maternal allele in brain tissue, the truncated variant *203*, which ends shortly after *Peg13* in intron 17, was identified as a paternal allele-specific transcript in RNA-seq studies (Gregg et al., 2010; Hsu et al., 2018). We set out to confirm these variants using RT-PCR across specific *Trappc9* exons. We readily detected the full-length transcript *202* in brain and other tissues of newborn mice but were not able to confirm the alternative first exon of the *201* transcript (Figure 1B). Exon 2 of the *202* transcript contains a conserved translational start codon across mammalian species. To further investigate potential alternative transcriptional start sites, we undertook 5′-RACE PCR and sequencing, which revealed several transcriptional start sites within the exon 1 5′-UTR of variant *202* (Figure 1C), but we did not detect the alternative exon 1 of variant *201*. Sequencing of PCR products also showed alternative splicing of exon 5, which was missing in some kidney and spleen cDNAs, in line with ENSEMBL annotations. Additionally, we investigated the alternative, truncated *Trappc9* splice forms *206* and *203*, which terminate in intron 17 upstream and downstream of *Peg13*, respectively (Figure 1A, Supplementary Figure S1). We were unable to detect any cDNA containing the shared exon 17 in combination with 3′-UTR exons of variants *206* and *203* in tissues (Supplementary Figure S1), or containing shared exon 16 in single neurosphere cells.

In conclusion, we have experimentally verified the transcriptional start sites of *Trappc9* located in exon 1 of the *202* variant as well as alternative splicing of exon 5 but found no evidence of an alternative promoter or truncated transcript variants.

### Allelic expression biases of the imprinting cluster genes in tissues and neurospheres

**FIGURE 2:**
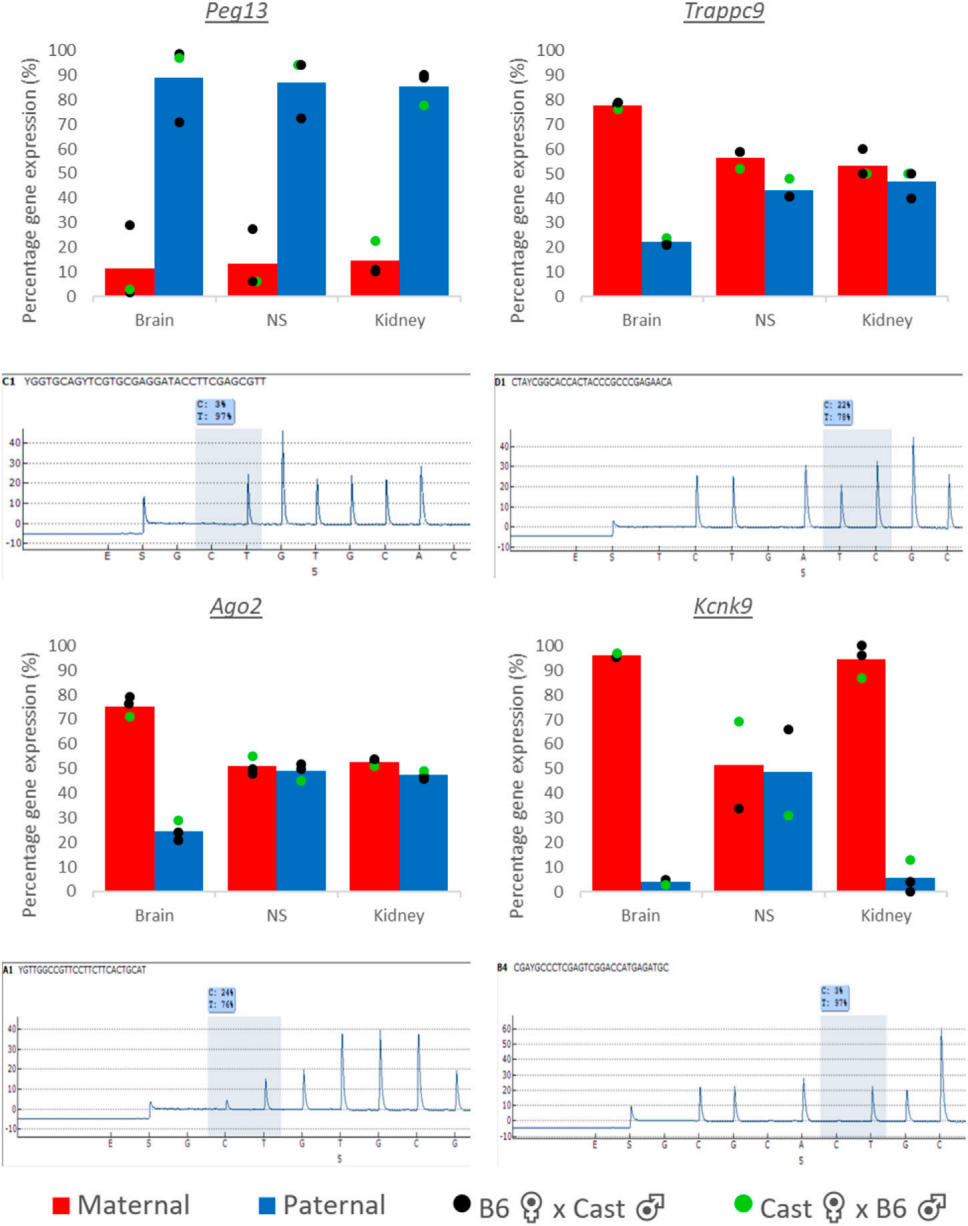
Allelic expression in tissues and primary hippocampal neurospheres of hybrid mice. Parental allelic expression was quantified *via* SNP pyrosequencing of cDNA from tissues or cultured neurospheres (NS) obtained from newborn mice. Average expression (*n* = 2–3) and example traces are shown. SNP IDs and exon locations are: *Peg13* (rs238259968 and rs31423566 located in exon 1), *Trappc9* (rs31440851 located in exon 2), *Ago2* (rs232384843 located in exon 5) and *Kcnk9* (rs225149059 located in exon 1). Points on the bar graphs represent individual pyrosequencing results from reciprocal crosses as shown in the legend. For *Peg13* each data point shows the average of the two SNP values in the same sequence read. Representative pyrograms from whole brain samples are shown, indicating the SNP position in the sequence and the quantification of allelic expression.

Apart from *Peg13*, which constitutes a canonical imprinted gene with mono-allelic paternal expression in a wide range of tissues, the other genes of the cluster have been characterized *via* RNA-seq as tissue-specifically imprinted with biased expression from the maternal allele in mouse brain (Babak et al., 2015; Perez et al., 2015; Andergassen et al., 2017). We set out to validate these findings through SNP pyrosequencing and additionally included samples of primary NSC (neurosphere) cultures from newborn mice (Supplementary Figures S2A,B), since some imprinted genes have been shown to become bi-allelically expressed specifically in postnatal and adult NSCs (Ferron et al., 2011; Montalban-Loro et al., 2021). We used tissues and cultured hippocampal neurospheres from reciprocal crosses of C57BL/6J and *Mus musculus castaneus* (Cast/EiJ) newborn F1 hybrids. While *Peg13* showed the expected, almost exclusive (80%–90%) expression from the paternal allele in brain, kidney and neurospheres, the other genes of this cluster displayed tissue-specific imprinted expression (Figure 2). *Trappc9* and *Ago2* were predominantly (70%–80%) transcribed from the maternal allele in brain, but showed equal bi-allelic expression in kidney, while *Kcnk9* was expressed almost exclusively (>90%) from the maternal allele in both tissues. Due to unavailability of SNPs between C57BL/6J and Cast/EiJ strains, we analyzed *Chrac1* in reciprocal crosses of C57BL/6J and *Mus musculus molossinus* (JF1) F1 tissue samples and found it to be bi-allelically expressed with only a small bias (<70%) towards the maternal allele in newborn brain (Supplementary Figure S3). We also confirmed brain-specific imprinted expression of *Trappc9* in these hybrids (Supplementary Figure S3). Unexpectedly and in contrast to brain tissue as a whole, *Ago2* and *Trappc9* were not imprinted in the NSC cultures, but showed equal bi-allelic or only slightly biased (<70%) expression (Figure 2), which is reminiscent of *Dlk1* (Ferron et al., 2011; Montalban-Loro et al., 2021). *Kcnk9* allelic expression in neurospheres was inconclusive and prone to strain-specific biases (Figure 2).

We undertook pyrosequencing and/or Sanger sequencing of bisulfite-treated DNA to address DNA methylation states at the CGIs of the genes in neurospheres. The germline differentially methylated region (DMR) at *Peg13* was maintained in NSCs with methylation observed on the maternal allele (Supplementary Figure S4A), in line with brain tissue observations (Ruf et al., 2007; Xie et al., 2012). The *Trappc9* CGI1, located at the promoter/exon1, was unmethylated in NSCs, brain and kidney (Supplementary Figure S4B), while the CGI2 at exon 2 was fully methylated on both alleles in NSCs and brain (Supplementary Figure S4C). The promoter CGIs at *Ago2*, *Chrac1* and *Kcnk9* were also unmethylated in NSCs (Supplementary Figures S5A−C), which is in line with human brain data (Court et al., 2014) and their status as actively transcribed genes. This excludes any secondary DMRs at this imprinted gene cluster.

Overall, our data confirm brain-specific imprinting, i.e., preferential expression from the maternal allele, of *Trappc9* and *Ago2* in mouse, while maternal allelic expression of *Kcnk9* occurs in brain and some peripheral tissues, e.g., kidney. Unexpectedly, *Trappc9* and *Ago2* have no allelic expression bias in hippocampal neurosphere cultures.

### Varying allelic expression biases of *Peg13*, *Trappc9* and *Ago2* in single neural stem cells and differentiated neurons

Analysis of imprinted gene expression on a bulk tissue level raises the question of whether the observed allelic bias is reflected in every cell of the lysate in the same way, or whether individual cells differ in their mono-/bi-allelic transcriptional status of the gene and, thus, deviate from the tissue average. Large-scale single-cell imprinted gene expression analysis is still in its infancy, but novel approaches indicate that not all cells of a tissue might show the same allelic expression status (Martini et al., 2022). To address this question specifically for the *Peg13* imprinting cluster, we isolated single NSCs from C57BL/6J × Cast/EiJ neurospheres as well as single neurons differentiated from these *in vitro*. We then undertook qRT-PCR using the sc-GEM (single-cell analysis of genotype, expression and methylation) technique (Cheow et al., 2016) and Sanger sequencing to determine allelic SNP expression for *Peg13*, *Trappc9* and *Ago2*. As a further confirmation of the NSC phenotype, we also verified marker gene expression (Hochgerner et al., 2018) in single neurosphere cells (Supplementary Figure S2C). Analyzing ∼50 NSCs and neurons, we found a variety of mono-allelic and bi-allelic expression states in individual cells.

**FIGURE 3:**
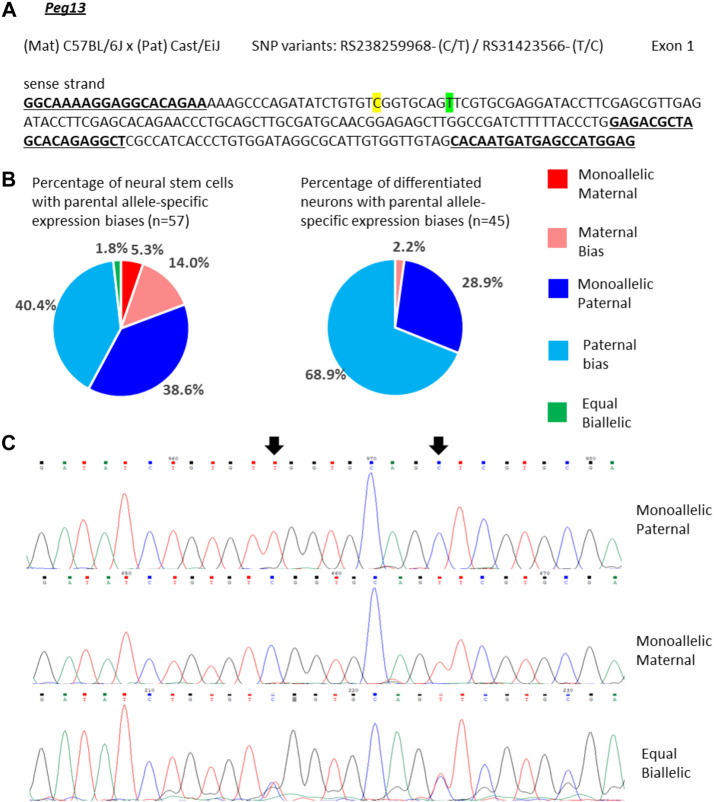
Variable single-cell allele-specific expression of *Peg13* analyzed through Sanger sequencing of cDNA SNPs from neural stem (neurosphere) cells and their *in vitro* differentiated neurons. **(A)** The mouse cross, cDNA amplicon and SNP locations in exon 1 are shown. B6 SNP variants are indicated in color; primers are underlined. **(B)** Summary data showing the proportions of cells falling into the five categories of allelic expression. **(C)** Example single-cell sequence tracks for the three expression categories indicated. Sequence tracks for cells with maternal or paternal bias displayed a major SNP peak for those alleles with a minor overlapping SNP peak for the other allele, respectively. SNP positions are highlighted by arrows.

For *Peg13*, 39% of the NSCs showed mono-allelic paternal expression and 40% of the cells had transcripts predominantly from the paternal allele, but additionally a small amount of maternal transcripts (Figures 3A–C). However, we also detected small numbers of cells with equal bi-allelic, predominantly maternal, or even mono-allelic maternal expression of *Peg13* (Figures 3B,C), which indicates a surprising heterogeneity of imprinted expression between individual NSCs. Proportionately, the 79% of cells with mono-allelic and biased paternal expression approximate the bulk neurosphere expression bias of 87% paternal transcripts (Figure 2). In *in vitro* differentiated neurons, the cellular heterogeneity was reduced as almost all cells showed mono-allelic or paternal bias of *Peg13* expression (Figure 3B), much in line with the paternal bias of 89% in brain tissue as a whole (Figure 2).

**FIGURE 4:**
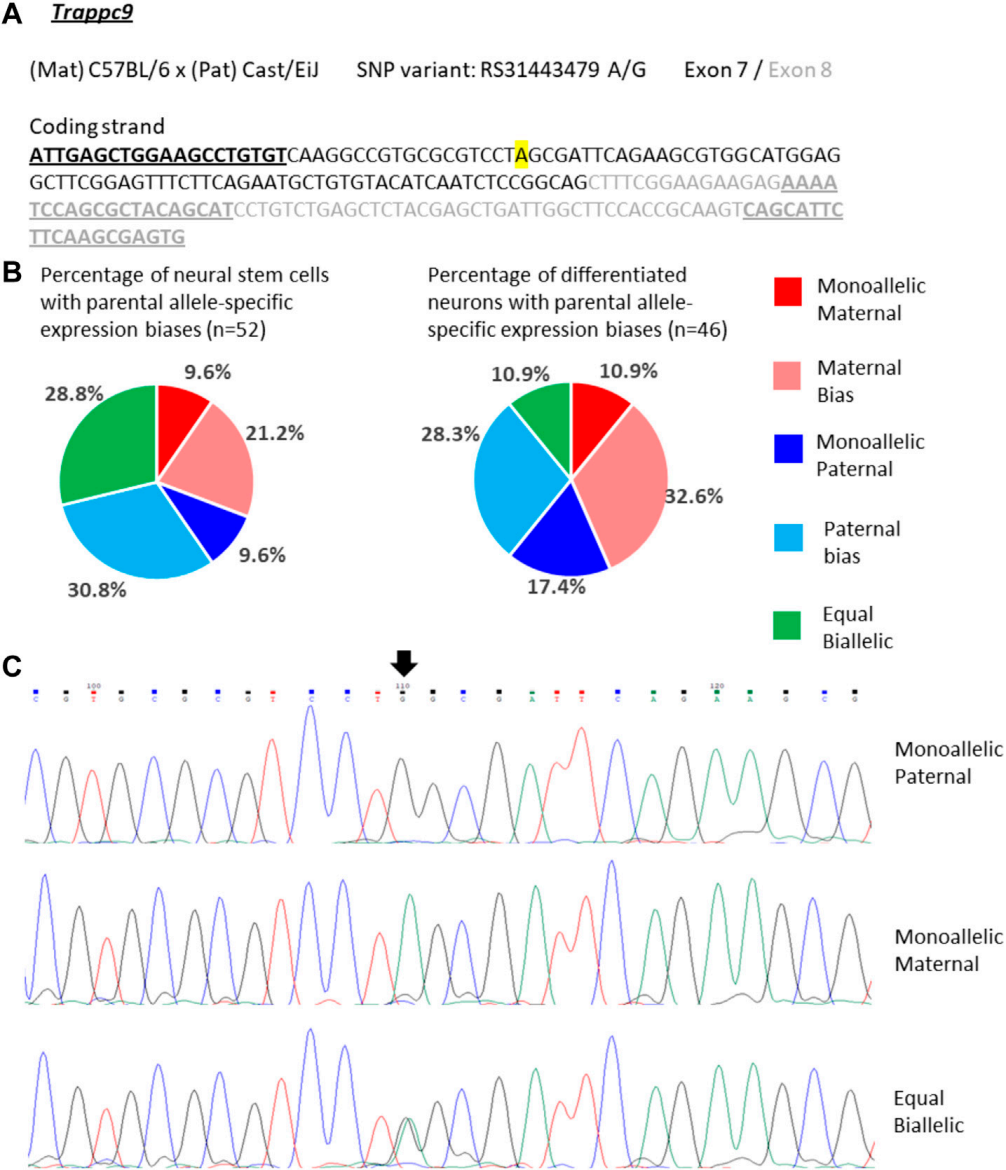
Variable single-cell allele-specific expression of *Trappc9* analyzed through Sanger sequencing of a cDNA SNP from neural stem (neurosphere) cells and their *in vitro* differentiated neurons. **(A)** The mouse cross, cDNA amplicon and SNP location in exon 7 are shown. B6 SNP variant is indicated in color; primers are underlined. **(B)** Summary data showing the proportions of cells falling into the five categories of allelic expression. **(C)** Example single-cell sequence tracks for the three expression categories indicated. Sequence tracks for cells with maternal or paternal bias displayed a major SNP peak for those alleles with a minor overlapping SNP peak for the other allele, respectively. SNP position is highlighted by an arrow.

For *Trappc9* the allelic expression varied considerably in single NSCs. We observed mono-allelic maternal or paternal *Trappc9* expression in 10% of the cells respectively, while 29% displayed equal bi-allelic transcription (Figures 4A–C). Around half of the NSCs showed biased bi-allelic expression: 21% predominantly maternal, 31% predominantly paternal (Figure 4B). Considering all single-NSC categories in proportion, the data are in line with the bulk neurosphere analysis (Figure 2) as no clear overall parental allele bias was observed in either dataset. In differentiated neurons, the proportion of cells with equal bi-allelic expression was reduced to 11%, while another 11% showed mono-allelic maternal and 17% mono-allelic paternal *Trappc9* expression (Figure 4B). Taking into account the proportions of neurons with biased bi-allelic expression (33% maternal, 28% paternal bias), the single-neuron dataset depicts an overall equal bi-allelic *Trappc9* expression, which is in contrast to the maternal expression bias of 78% that was observed in whole-brain lysate (Figure 2). This discrepancy might be due to the brain lysate containing additional cell types (e.g., astrocytes, microglia, oligodendrocytes) and a wider range of neuron types from different brain sub-regions as compared to the *in vitro* differentiated neurons, which were derived from hippocampal neurospheres.

**FIGURE 5:**
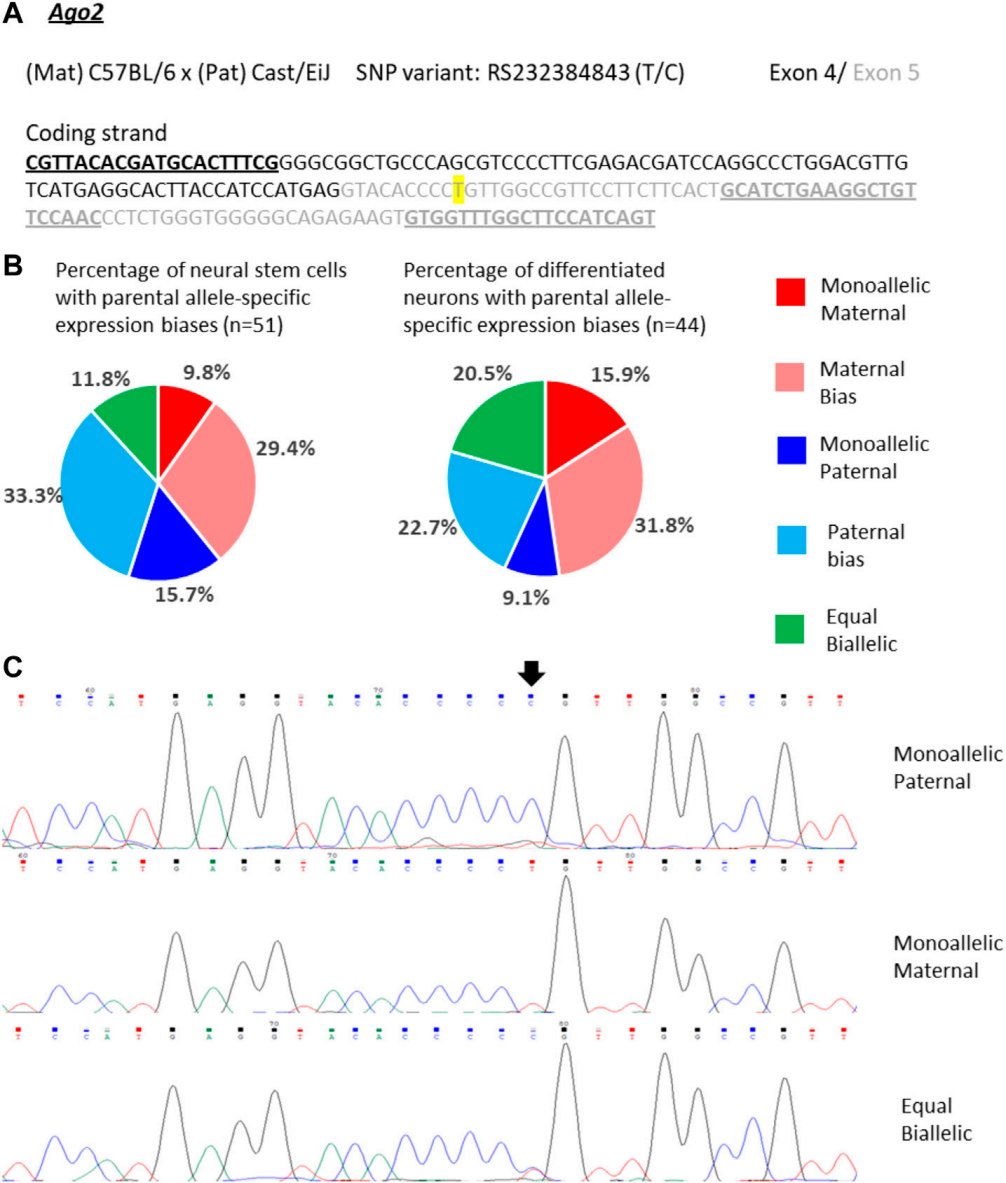
Variable single-cell allele-specific expression of *Ago2* analyzed through Sanger sequencing of a cDNA SNP from neural stem (neurosphere) cells and their *in vitro* differentiated neurons. **(A)** The mouse cross, cDNA amplicon and SNP location in exon 5 are shown. B6 SNP variant is indicated in color; primers are underlined. **(B)** Summary data showing the proportions of cells falling into the five categories of allelic expression. **(C)** Example single-cell sequence tracks for the three expression categories indicated. Sequence tracks for cells with maternal or paternal bias displayed a major SNP peak for those alleles with a minor overlapping SNP peak for the other allele, respectively. SNP position is highlighted by an arrow.

*Ago2* expression in single NSCs was similarly variable as *Trappc9* expression. 10% of the NSCs displayed mono-allelic maternal, 16% mono-allelic paternal and 12% equal bi-allelic expression (Figures 5A–C). Most NSCs had a biased bi-allelic expression of *Ago2* (29% predominantly maternal, 33% predominantly paternal). Overall, the proportions of NSCs falling into the various expression categories did not indicate a clear allele bias and are in line with the equal bi-allelic *Ago2* expression found in bulk neurosphere samples (Figure 2). Compared to the NSCs, among the differentiated neurons more cells showed equal bi-allelic (21%), mono-allelic maternal (16%) and biased maternal (32%) expression (Figure 5B), while the proportions of neurons with mono-allelic paternal (9%) and biased paternal (23%) expression were reduced (Figure 5B). However, as with *Trappc9*, the *in vitro* differentiated neuron categories did not overall reflect the same strong bias of 75% maternal allele-specific *Ago2* transcripts that were detected in whole-brain tissue (Figure 2).

Since the non-coding RNA *Peg13* is transcribed from the core imprinting regulatory region (DMR) of the locus, findings of *Peg13* expression other than mono-allelic paternal or paternally biased bi-allelic (Figures 3B,C) were unexpected. This raises the question of whether there is a specific pattern of allelic expression of the other imprinted genes of the locus associated with *Peg13* transcription from the maternal allele. When analyzing the expression status of *Trappc9* and *Ago2* in those cells that showed equal bi-allelic, maternally biased or mono-allelic maternal expression of *Peg13*, we did not find any specific patterns or correlations (Supplementary Table S2). Some of these cells displayed the expected maternal bias of *Trappc9* and/or *Ago2*, but other allelic biases, including mono-allelic expression states, were also observed and in varying combinations within individual cells.

In summary, our single-cell analysis of the three imprinted genes indicates a surprising variability of allelic expression states in individual cells, ranging from mono-allelic maternal to mono-allelic paternal transcription, even for the core imprinted gene of the locus, *Peg13*. Overall, *Peg13* transcriptional states in the NSC and neuron populations matched the brain tissue level of allelic expression (∼89% paternal) very well as most cells displayed mono-allelic or strong paternal bias. *Trappc9* and *Ago2*, which represent tissue-specifically imprinted genes with a maternal expression bias of ∼75% in brain, showed much more variability in their allelic transcriptional states in individual NSCs and neurons; all categories of allelic transcription were represented by substantial numbers of cells. Thus, our data do not support a model, in which a tissue-level imprinted gene expression status is reflected in each cell of the tissue in the same way. These findings might hint at a certain level of transcriptional noise or transient/random bursts of transcription, which might still be able to occur at alleles that are “silenced” by genomic imprinting (Varrault et al., 2020).

### Analysis of potential gene regulatory regions indicates several silencer elements for *Trappc9*

**FIGURE 6:**
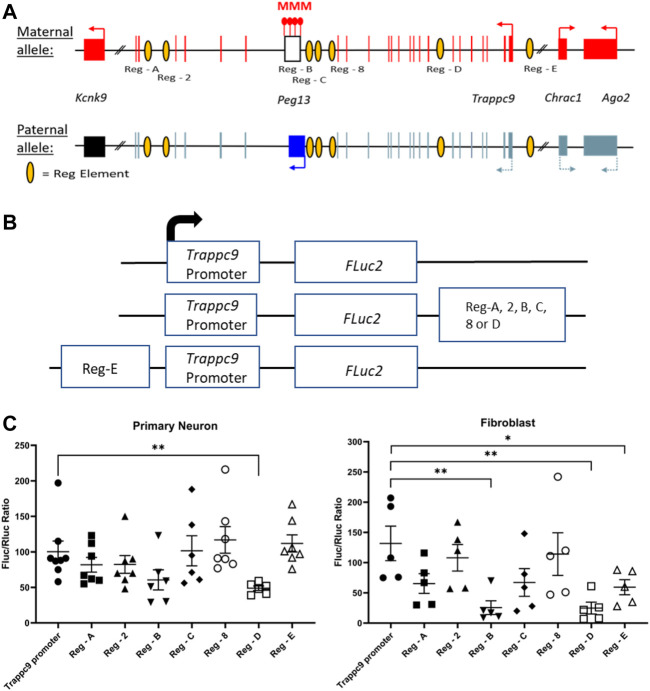
Promoter-reporter gene assays indicate silencer elements for *Trappc9*. **(A)** Scheme of the murine *Peg13—Kcnk9—Trappc9* cluster of imprinted genes. For *Trappc9* all introns are shown; for *Chrac1*, *Ago2* and *Kcnk9* introns have been omitted. Promoters, transcriptional activity and directions are indicated by arrows. Also indicated are the approximate locations of the candidate regulatory elements (listed in Supplementary Figure S6) across *Trappc9* introns. Not to scale. **(B)** Schematic of the promoter/enhancer constructs used for transfection of primary cells. Enhancers were positioned downstream or upstream of the promoter-reporter gene cassette depending on their relative locations within the *Trappc9* gene and in the same orientation. **(C)** Reporter gene activity of the constructs in cultures of primary hippocampal neurons from newborn mice and primary embryonic fibroblasts. Normalized activity of Firefly luciferase (FLuc) to co-transfected Renilla luciferase (RLuc) is shown. Mean values ± S.E.M. are indicated. *p ≤ 0.05; **p ≤ 0.01.

It is currently unclear how tissue-specific imprinting and maternal allele-biased expression of *Trappc9* and *Ago2* are regulated in the mouse brain. Furthermore, imprinting of the two genes is not conserved in humans (Court et al., 2014) and only homozygous mutations of *TRAPPC9* cause a neurodevelopmental disorder, which is characterized by intellectual disability, speech impairment and microcephaly (Wilton et al., 2020; Aslanger et al., 2022). A potential mechanism could involve chromatin boundaries and CTCF-regulated access to tissue-specific enhancers as has been shown for the imprinted *Igf2-H19* locus (Bell and Felsenfeld, 2000). Indeed, CTCF binding on the unmethylated paternal allele of the *Peg13* DMR has been demonstrated in mouse brain and fibroblasts (Singh et al., 2011; Prickett et al., 2013) as well as in human brain (Court et al., 2014). In humans, CTCF regulates access of *KCNK9* and *PEG13* promoters to a brain-specific enhancer, most likely in a differential, allele-specific way (Court et al., 2014). However, since the imprinting status of the genes upstream of *Peg13*, i.e., *Trappc9*, *Ago2* and *Chrac1*, differs between mouse and human brain, their regulation presumably involves enhancers that are not conserved or additional mechanisms. We, therefore, screened Encode3 mouse genome data for chromatin modifications and accessibility (Consortium et al., 2020; Gorkin et al., 2020) across the *Trappc9-Peg13* locus in the UCSC Genome Browser and identified seven potential brain-specific regulatory elements with appropriate histone modification (H3K4 methylation, H3K27 acetylation, H3K9 acetylation), ATAC and DNAse I hypersensitivity marks (Supplementary Figure S6). To test these candidate elements in promoter-reporter gene assay, we first constructed a *Luciferase* plasmid that contained a 444 bp *Trappc9* promoter fragment upstream of and including non-coding exon 1 (Figure 1C). The size of this promoter fragment is in line with standards from high-throughput testing of promoter-enhancer interactions in the mouse genome (Martinez-Ara et al., 2022). We positioned the candidate regulatory elements downstream or upstream of the promoter-reporter gene cassette, depending on their relative locations within the *Trappc9* locus (Figures 6A,B). We performed reporter gene assays in cultures of mouse primary hippocampal neurons and embryonic fibroblasts. Compared to the promoter-only construct, regulatory elements Reg-B and Reg-E had significant silencing effects in fibroblasts, but not neurons, indicating a tissue-/cell type-specific function (Figure 6C). Reg-D displayed silencing activity in both neurons and fibroblasts. Unexpectedly, none of the regulatory elements showed enhancer activity in our assay conditions. All three silencing elements are located on the *Trappc9*-proximal side of the *Peg13* DMR and CTCF-binding site (Figure 6A). These silencer elements might contribute to the regulation of tissue-specific expression of *Trappc9*, *Chrac1* and/or *Ago2 in vivo*. Reg-D might also contribute specifically to the reduced transcription of their paternal alleles in brain, thereby generating an imprinted expression bias in this tissue, although any allele-specific mechanism remains to be elucidated.

## Discussion

Our findings of allelic expression biases of these imprinting cluster genes in newborn mouse brain are in line with previous data from whole transcriptome studies, showing strong (∼90%) paternal and maternal preferences for *Peg13* and *Kcnk9*, respectively, while *Trappc9* and *Ago2* displayed a more moderate (∼75%) preference for the maternal allele (Babak et al., 2015; Bonthuis et al., 2015; Crowley et al., 2015; Perez et al., 2015; Bouschet et al., 2016; Andergassen et al., 2017; Huang et al., 2017). *Chrac1* fell below the threshold of 70% bias in our data and also showed a weaker maternal bias than *Trappc9* and *Ago2* in the studies by Crowley et al. (2015) and Perez et al. (2015). Brain-specificity of *Trappc9* and *Ago2* imprinting was also confirmed, since we found expression in kidney to be equal bi-allelic, in line with previous data. Unexpectedly, we did not detect an imprinted expression bias for *Trappc9* and *Ago2* in cultured hippocampal NSCs (neurospheres), where both genes showed equal bi-allelic expression in bulk sample analysis. By contrast, *Peg13* retained its strong paternal expression bias in neurospheres. These findings are reminiscent of another imprinted gene, *Dlk1*, which loses its mono-allelic paternal expression and becomes bi-allelically expressed in postnatal NSCs of the ventricular zone and hippocampal subgranular zone (Ferron et al., 2011; Montalban-Loro et al., 2021). The change to bi-allelic expression of *Dlk1* is associated with gain of methylation at its germline DMR and is a requirement for normal postnatal and adult neurogenesis. However, we did not find any change in methylation at the *Peg13* germline DMR in neurospheres. Also, the promoter CGI of *Trappc9* remained unmethylated on both alleles, while the second CGI at exon 2 retained its high levels of methylation in neurospheres. Similarly, the *Ago2* promoter CGI remained unmethylated on both alleles in neurospheres. Thus, the regulation of allelic expression of *Trappc9* and *Ago2* in NSCs is likely to differ from that in differentiated neural cells and might involve changes in histone modifications, transcription factor binding and/or enhancer access. In any case, a relevance of *Trappc9* expression in NSCs is implied by the finding of reduced numbers of Sox2-positive stem cells in the subventricular zone and hippocampal subgranular zone of knock-out mice (Usman et al., 2022), which might be linked to their microcephaly phenotype (Ke et al., 2020; Liang et al., 2020; Wilton et al., 2020; Aslanger et al., 2022).

Due to advances in technology, especially single-cell transcriptomics and highly sensitive *in situ* hybridization methods, it has now become possible to investigate imprinted gene expression on the cellular level (Varrault et al., 2020; Martini et al., 2022). Instead of a single-cell RNA-seq approach, we used the sc-GEM method (Lorthongpanich et al., 2013; Cheow et al., 2015; Cheow et al., 2016) for a more limited analysis of the genes of this imprinting cluster in single cultured NSCs and differentiated neurons. Our data for *Trappc9* and *Ago2* show a broad variability of allelic expression status in individual neurosphere cells. All categories of allelic expression, ranging from mono-allelic maternal to equal bi-allelic to mono-allelic paternal and intermediately biased bi-allelic states, were found in significant numbers of cells. Taking into account all single NSCs analyzed, the allelic expression of these two genes leveled out in line with the bulk neurosphere data, i.e., overall there was no allelic bias in the neural stem cell population. Our findings were similar in single neurons that were differentiated from the neurospheres; significant numbers of cells were found for each category of allelic expression. When comparing the cell population of NSCs with the neuronal population for *Ago2* expression biases, a slight overall shift from paternal to maternal allelic biases was observed. For *Trappc9*, the proportion of cells with equal bi-allelic expression was reduced in the neuron population compared to the NSC population, but the proportions of cells with maternal and paternal expression biases, respectively, remained balanced. Neither of the two genes displayed an overall maternal expression preference in the population of single neurons, which is in contrast to the data obtained from brain lysates. However, our neuron culture is not fully representative of all the cell types that would be included in a brain tissue lysate, since we actively selected against dividing glial cells by adding Ara-C to the culture. Furthermore, since the neurons were differentiated from hippocampal NSCs, our culture is likely to contain only a limited range of neuronal cell types. Data from the Allen Brain Map (https://portal.brain-map.org/) *in situ* hybridization atlas and single-cell transcriptomics indicate medium levels of *Trappc9* and *Ago2* expression in many neurons of the cortex and hippocampus, with lower levels occurring in some types of neurons as well as astrocytes and oligodendrocytes. Limited histological analysis for *Trappc9* by Ke et al. (2020) support these data.

On a more general note, we have no indication that our single-cell data are affected by potential technical issues, for example, allele drop-out during reverse transcription, and we have not found a way to test for such eventualities at extremely low numbers of RNA molecules. However, this would affect the weakly expressed allele, i.e., the paternal allele of *Trappc9* or the maternal allele for *Peg13* as judged from brain lysates, more than the strongly expressed allele and should lead to an increased number of cells with mono-allelic maternal expression of *Trappc9*, or exclusively cells with mono-allelic paternal expression of *Peg13*. There is no indication for such an effect in our data. On the contrary, our data show the opposite, i.e., a surprisingly large number of cells that display a biased paternal expression of *Peg13* with weak expression of the maternal allele readily detectable, and even cells with predominantly maternal *Peg13* expression. Such results would not be expected, if there were a significant rate of allelic drop-out of the weakly expressed allele. Furthermore, our findings of varying mono- or bi-allelic expression states of *Ago2* in individual neurons is in line with *in situ* hybridization data obtained by Bonthuis et al. (2015), who analyzed nascent transcripts in nuclei of brain sections and determined that 46% of *Ago2* expressing cells in the arcuate nucleus, and 63% in the dorsal raphe nucleus, showed mono-allelic expression with the remainder having two visible sites of nuclear transcription.

For *Peg13*, our single-cell data indicated a predominantly paternal expression bias in NSCs, and even more so in differentiated neurons, although there was a substantial proportion of cells with paternally biased bi-allelic (instead of mono-allelic) expression. A small number of cells, mainly NSCs, deviated from this expected bias and showed equal bi-allelic or even mono-allelic maternal expression. Although surprising, these findings are not unprecedented. A recent study of imprinted gene expression in single cortical cells identified similar variability and occasional deviations from expected biases (Laukoter et al., 2020). For example, *Meg3* (also known as *Gtl2*), which usually has a strong maternal expression bias, was found to be bi-allelic in a small number of cortical cells. For two other imprinted genes with an expected paternal expression bias, i.e., *Inpp5f* and *Impact*, a substantial number of cortical cells deviated towards bi-allelic or predominantly maternal expression (Laukoter et al., 2020). The mechanisms and reasons behind such variable allelic expression states of imprinted genes in individual cells are largely unclear. The cases of *Dlk1* gain of methylation and loss of imprinting in NSCs (Ferron et al., 2011; Montalban-Loro et al., 2021), or *Grb10* alternative promoter usage on the maternal and paternal alleles (Yamasaki-Ishizaki et al., 2007; Sanz et al., 2008; Garfield et al., 2011) are unlikely models for our findings. On the other hand, random mono-allelic expression (RMAE) effects, especially transcriptional bursting (Reinius and Sandberg, 2015; Chess, 2016; Xu et al., 2017; Varrault et al., 2020), might affect imprinted genes and be the underlying reason for the variable allelic expression states we find in single cells. Such RMAE effects might be stochastic and dynamic, rather than permanent as in the case of random allelic exclusion of immunoglobulin genes, and might involve relatively short-lived CTCF-cohesin chromatin loops (Gabriele et al., 2022).

For the human *PEG13-KCNK9* locus, CTCF-cohesin binding sites, chromatin looping and enhancer interactions with those two gene promoters have been described (Court et al., 2014). CTCF-binding on the unmethylated paternal allele of the *Peg13* gDMR is conserved in mice (Singh et al., 2011; Prickett et al., 2013), but tissue-specific enhancer elements and chromatin looping might differ in this species and could underly the imprinted expression of *Trappc9*, *Chrac1* and *Ago2* in murine brain tissue. Our ENCODE3-based search for brain-specific regulatory elements considered active histone and open chromatin marks and resulted in several candidate regions, but unexpectedly these did not show enhancer function when tested in transfected primary neurons or fibroblasts. Instead, two elements had silencing activity specifically in fibroblasts, while a third element silenced reporter gene activity in both neurons and fibroblasts. Typically, active enhancers are associated with active chromatin marks, e.g., H3K27ac, H3K4me1, H3K9ac, and silencers are variably marked by H4K20me, H3K9me3 (typical for heterochromatin and methylated DNA) and/or H3K27me3, although chromatin at silencer regions is still expected to be open for binding of repressive transcription factors and, therefore, also associated with H3K79me2 and H3K36me3 marks (Pang and Snyder, 2020). However, there is currently no widely accepted consensus for a silencer chromatin signature and many silencer elements might be bifunctional elements acting through various mechanisms (Segert et al., 2021). Furthermore, a recent functional study of ENCODE3 candidate cis-regulatory elements (cCREs) found that the majority of annotated cCREs had no effect on transcription, while similar numbers of the remaining elements had enhancer or repressor activity, respectively, which was surprising given that cCREs are predicted to be enhancers (Martinez-Ara et al., 2022). Further experiments will be required to determine whether the silencer elements we identified within the *Trappc9* locus might function in an allele-specific way and contribute to the brain-specific imprinted expression bias of this gene.

A number of *Trappc9* transcript variants have been annotated on ENSEMBL, including two alternative promoters and truncated transcripts, one of which has been described as specifically expressed from the paternal allele in RNA-seq data (Gregg et al., 2010; Hsu et al., 2018). Our attempts to confirm such truncated transcript versions with primer combinations that span transcript-specific and shared exons were unsuccessful. *Trappc9* and *Peg13* are transcribed in the same direction and *Peg13* is an unspliced long non-coding RNA located in intron 17 of *Trappc9*. *Peg13* transcription from the paternal allele might extend further downstream than is currently known, similar to other long non-coding RNAs at imprinted loci, e.g., *Nespas*, or *Meg3/Gtl2* (Ferguson-Smith, 2011; Peters, 2014). Therefore, allelic expression analysis of *Trappc9* RNA downstream of the *Peg13* start site requires careful consideration. We also found no evidence for a second promoter of *Trappc9.* The transcriptional start sites we identified at the first non-coding exon will create transcripts with a translational start site in exon 2, which encodes the well-conserved NH_2_-terminal end of the protein. This would be missing upon alternative promoter usage. From our data, we can exclude a second promoter, which is also of relevance in the context of the variable allelic expression of *Trappc9* discussed above. We can exclude a second promoter as a possible explanation for the variable allelic expression in single cells.

Overall, the mechanisms of brain-specific imprinted expression of *Trappc9* in mice remain to be fully elucidated, but this allelic bias is of biological relevance, since maternal transmission of a knock-out mutation of *Trappc9* results in phenotypes similar to homozygous deletion and, *vice versa*, mice carrying a paternally transmitted mutation are not different from wild-types (Liang et al., 2020).

## Data Availability

The datasets presented in this study can be found in online repositories. The names of the repository/repositories and accession number(s) can be found below: https://zenodo.org/record/7002711#.YwSFdnbMKUk.
