# Supplementary material for: Variable allelic expression of imprinted genes at the Peg13, Trappc9, Ago2 cluster in single neural cells
Source: Front Cell Dev Biol. 2022 Oct 12;10:1022422. doi: 10.3389/fcell.2022.1022422 (PMC9596773; doi:10.3389/fcell.2022.1022422)
Supplement: Supplementary file 1 [file DataSheet7.PDF]

Supplementary Table S1: Primers used in experiments. Chromosome positions refer to the mouse GRCm38/mm10 genome version.

Primer Name	Sequence	Position
**Trappc9 Variant detection**		
Pr_02 Fw	TCACAGCGTGCACTCTTCAT	Trappc9 Exon 2 (Rat)
Pr_04 Fw	GTTGTTGGGTCTGAAAAGAC	Trappc9 Exon 2 (Rat)
Pr_04 Rv	GGGTCAATGAGAACTTCTTGC	Trappc9 Exon 5 (Rat)
Pr_05 Rv	GCTGGTGTCAGGATTTATGC	Trappc9 Exon 6 (Rat)
Pr_06 Fw	CAGACCCACAAACCCTTCT	Trappc9 Exon 17
Tr_203-RV	CTTTATTCACACTGTCCCTGA	Trappc9 variant 206 - last exon and 3′-UTR
Tr_204-RV	CTGCTAAGGAAAGACGCACA	Trappc9 variant 203 - last exon and 3′-UTR
Tr_203-F1	GCAGATAGCCATGCACCTT	Trappc9 intron upstream of variant 206 last exon and 3′-UTR
Tr_204-F1	AGTGGCAGCTGTTTAATGTC	Trappc9 intron 17 upstream of variant 203 last exon and 3′-UTR
**Pyrosequencing**		
Ago2 Meth F1	ATTAGTATTTGAATGGGGA	Ago2 CGI
Ago2 Meth R1B	**(Btn)** CATAACTATAAAACCCAACAC	
Ago2 Meth S1	GTATTTGAATGGGGAGG	
Chrac1 Meth F1B	**(Btn)**GGAGGAGGAGGTGTGTAG	Chrac1 CGI
Chrac1 R1	CCTTAACCTTTAACTACCCTAC	
Chrac1 Meth S1	CCTTAACCTTTAACTACCC	
Kcnk9 Meth F1	GAGGTGTTTAGGATTAGATAGTT	Kcnk9 CGI
Kcnk9 Meth R1B	**(Btn)**CCTATACCAAACCTAAATCAA	
Kcnk9 Meth S1	GGTGTTTAGGATTAGATAGT	
Peg13 Meth F1	AGGTTTTGTGTGATAGTTTATTTAAG	Peg13 CGI
Peg13 Meth R1B	**(Btn)** TCTTCTATCCAACCATTTTCA	
Peg13 Meth S1	GTTGGTGTTATGTAGA	
Trappc9 CGI1 Meth F1	GGTAGAGGGATTGAGTAGTTTG	Trappc9 CGI1
Trappc9 CGI1 Meth R1B	**(Btn)** CCCCAAACAAATCTTAAACC	
Trappc9 CGI1 Meth S1	GGGATTGAGTAGTTTGGA	
Trappc9 CGI2 Meth F3	GTAGGGTGATGTGGTTGAG	Trappc9 CGI2
Trappc9 CGI2 Meth R3B	**(Btn)**ACCCTTAAACTCCAACACAAT	
Trappc9 CGI2 Meth S3	GGTGATGTGGTTGAGTA	
Ago2 snp F1	CGTTGTCATGAGGCACTTACC	Ago2 Exon 4
Ago2 snp R1B	**(Btn)** GTTGGAACAGCCTTCAGATGC	Ago2 Exon 5
Ago2 snp S1	CCATGAGGTACACCCC	Ago2 Exon 4/5
Chrac1 snp F77B	**(Btn)** AAGCCAAGAAAGCACTGAC	Chrac1 Exon 2
Chrac1 snp R77	AATATATCTGCGAGAAACTGAAG	Chrac1 Exon 3

Chrac1 snp S9	GAGAAACTGAAGTGTCTCC	Chrac1 Exon 2
Kcnk9 snp F1	AACGTGCGTACCCTGTCCT	Kcnk9 Exon 1
Kcnk9 snp R6B	**(Btn)** CTCCTCGCGCATCTCATG	Kcnk9 Exon 1
Kcnk9 snp S1	CGGTGCCGCGGTGTT	Kcnk9 Exon 1
Peg13 snp F53	GGCAAAAGGAGGCACAGAA	Peg13 Exon 1
Peg13 snp R53B	**(Btn)** GCTGCAGGGTTCTGTGCTC	Peg13 Exon 1
Peg13 snp S1	AAGCCCAGATATCTGTGT	Peg13 Exon 1
Trappc9 snp F1	CAGCGTGCCCTCTTCATCC	Trappc9 Exon 2
Trappc9 snp R3B	**(Btn)** TGCGGTGCGTCTGGAAGT	Trappc9 Exon 2
Trappc9 snp S1	TGCCCTCTTCATCCG	Trappc9 Exon 2
Trappc9_snp1 Fb	**(Btn)** CAGCAAGTACAAGAACGCCG	Trappc9 Exon 7
Trappc9_snp1 R	CTCCATGCCACGCTTCTG	Trappc9 Exon 7
Trappc9_snp1 S	CGCTTCTGAATCGCTAGG	Trappc9 Exon 7
Trappc9_snp2 Fb	**(Btn)** AAGGACTTCAGCAAAGGCACA	Trappc9 Exon 9
Trappc9_snp2 R	ATCCTGCGGGAGGCATAGA	Trappc9 Exon 9
Trappc9_snp2 S	ATAGACCAGCTCATGCA	Trappc9 Exon 9
**Bisulfite Sanger sequencing analysis**		
Ago2 Full CpG island F1	TTTATAGTGAAGAAGTTTGGGGAG	Ago2 CGI
Ago2 Full CpG island R1	TAACAACTAATAAAATTTAATCCTAAAC	
Chrac1 Full CpG island F1	TGAAAGGATAAATTTGTGTAGT	Chrac1 CGI
Chrac1 Full CpG island R1	AAACCCTAAACAACCTTACAAAC	
Kcnk9 Full CpG island F1	TTATTAGTTGGTTGGGGA	Kcnk9 CGI
Kcnk9 Full CpG island R1	AAAAATAAAATCATACCCCTAAAAA	
Peg13 Full CpG island F1	GGGGTTTTATTGTGTGGG	Peg13 CGI
Peg13 Full CpG island R1	CTCCATAACTCATCATTATACTACAACCA	
Trappc9 Full CGI1 F1	AGATAGAGGATTAGGTAAGTAGGGGG	Trappc9 CGI1
Trappc9 Full CGI1 R1	TAATCTTCTACTCCAAAATCTACCAA	
CpG1_02Fw	GGTGGTTTGGAGGTTTTAGGTTGTTTAG	Trappc9 CGI1
CpG1_02Rv	CCAAACAACTAACAACAAAATAACAAACT ATCC	Trappc9 CGI1
Trappc9 Full CGI2 F1	TGTGAGTTTTGTAAGGTAGAGA	Trappc9 CGI2
Trappc9 Full CGI2 R1	CTTAAACTCCAACACAATAAAAA	
CpG2_01Fw	AGAGTGTGGTATGTTTTTGTTTATTAGTG TTAATG	Trappc9 CGI2
CpG2_01Rv	AACTTCTCAAAAATCTATAACCAATCTTTA AAC	Trappc9 CGI2
**Sc-GEM**		
B actin expression F	AAGGCCAACCGTGAAAAGAT	Exon 3
B actin expression R	GTGGTACGACCAGAGGCATAC	Exon 4
Ago2 cDNA F	CGTTACACGATGCACTTTCG	Exon 4
Ago2 cDNA Nested R	GTTGGAACAGCCTTCAGATGC	Exon 5
Ago2 cDNA Outer R	ACTGATGGAAGCCAAACCAC	Exon 5
Peg13 cDNA F	GGCAAAAGGAGGCACAGAA	Exon 1
Peg13 cDNA Nested R	AGCCTCTGTGCTAGCGTCTC	Exon 1
Peg13 cDNA R	CTCCATGGCTCATCATTGTG	Exon1
Trappc9 cDNA F	ATTGAGCTGGAAGCCTGTGT	Exon 7

Trappc9 cDNA Nested R	ATGCTGTAGCGCTGGATTTT	Exon 8
Trappc9 cDNA Outer R	CACTCGCTTGAAGAATGCTG	Exon 8
Aqp4 F	AGCAATTGGATTTTCCGTTG	Exon 2
Aqp4 Nested F	CTGGGCAAACCACTGGATAT	Exon 3
Aqp4 R	TGAGCTCCACATCAGGACAG	Exon 4
Wnt8b F	ACTCCCGAAATGGACAACTG	Exon 1
Wnt8b Nested F	GAGAGGCAATTTCCAAGCAG	Exon 2
Wnt8b R	TTACACGTGCGTTTCATGGT	Exon 3
Cdk1 F	CTGGGCAGTTCATGGATTCT	Exon 4
Cdk1 Nested F	ATCAAACTGGCTGATTTCGG	Exon 5
Cdk1 R	GATGTCAACCGGAGTGGAGT	Exon 6
Eomes F	GGCCTACCAAAACACGGATA	Exon 4
Eomes Nested F	TTCCGGGACAACTACGATTC	Exon 5
Eomes R	GTCACGTCAACTTCACAGCA	Exon 5
Mxd3 F	CCAGGGTGCATATCCAGAAG	Exon 3
Mxd3 Nested F	CTTCAGGCCTGTCCTCTGAG	Exon 4
Mxd3 R	CACATCCACCTCCAGATCCT	Exon 5
Calb2 F	TGGCGGAAGTATGACACAGA	Exon 5
Calb2 Nested F	CTCCTGAAGAAGGCCAACAG	Exon 6
Calb2 R	CCAATTTGCCATCTCCATTT	Exon 7
Igfbpl1 F	CCCTTCTGACCATGAGACCA	Exon 2
Igfbpl1 Nested F	AGGGAGTGTACCACTGCCAC	Exon 3
Igfbpl1 R	CACCCGGAACTGAGGAGTAG	Exon 3
Nestin F'	GATCGCTCAGATCCTGGAAG	Exon 2
Nestin Nested F'	TGAGAACTCTCGCTTGCAGA	Exon 3
Nestin R'	AGAGAAGGATGTTGGGCTGA	Exon 4
**Enhancer-Luciferase assay**		
Trappc9 Promoter region F2	CAGACCCTGGTGATAGCTT	Chr15:72,932,050 – 72,933,649
Trappc9 Promoter region R1	CCAAGCAGGAGCCGAG	
Regulatory element- A F1	TAGATCAGACGCAGGACACA	chr15:72625401-72626800
Regulatory element- A R1	ACTTTGAAACTTCCCTCTTC	
Regulatory element- B F1	AGGAACTGACCCATAGGAGT	chr15:72849700-72853300
Regulatory element- B R1	CTGGGATTTGAACTCTGGA	
Regulatory element- C F1	TCACCTCTTCAAGACTCCAT	chr15:72860700-72862300
Regulatory element- C R1	CAATGGCATTCCAAACCAA	
Regulatory element- D F1	TGCACCACTCTAGCAATCTT	chr15:73019700-73023500
Regulatory element- D R1	GTCCAGGGAAGCCTTGAA	
Regulatory element- E F1	GCCCAATACCCAACAGCAG	chr15:73066701-73068500
Regulatory element- E R1	GGTTTCCTGTGTCGTTCCG	
Regulatory element- 2 F1	ACGAATGTGACCTCTCCTCC	chr15:72657300-72658700
Regulatory element- 2 R1	CCTTAGTTGTGCACACCTACA	
Regulatory element- 8 F1	GGAGGCACACAGAACCACA	chr15:72,891,601-72,892,600
Regulatory element- 8 R1	CCTCTCAAGAGCTCTAGTGAT	
