# Supplementary material for: Variable allelic expression of imprinted genes at the Peg13, Trappc9, Ago2 cluster in single neural cells
Source: Front Cell Dev Biol. 2022 Oct 12;10:1022422. doi: 10.3389/fcell.2022.1022422 (PMC9596773; doi:10.3389/fcell.2022.1022422)
Supplement: Supplementary file 2 [file DataSheet2.PDF]

A

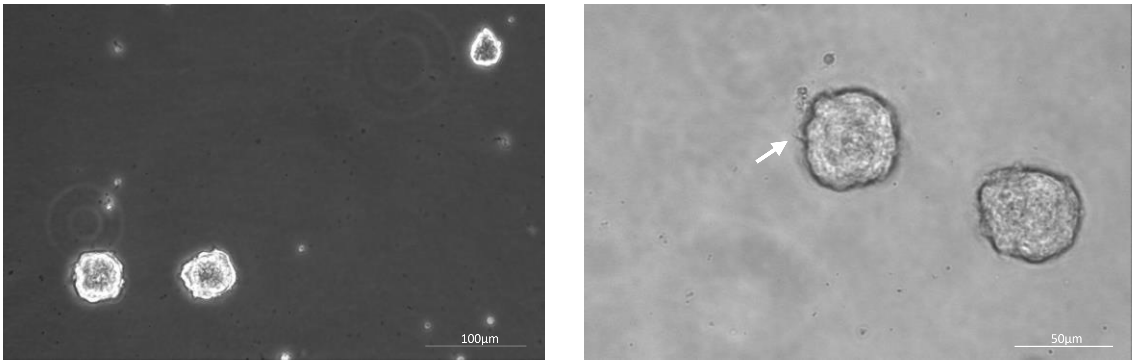

B

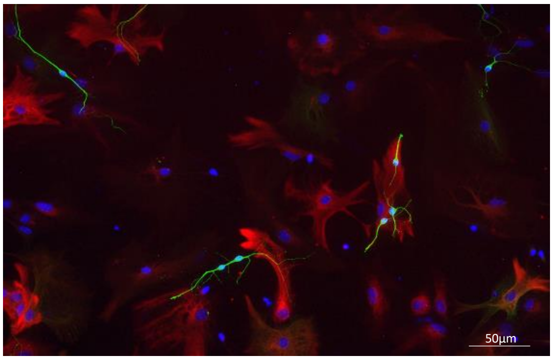

C

Cell markers	Cell 1	Cell 2	Cell 3	Cell 4	Cell 5	Cell 6	Cell 7	Cell 8	Cell 9	Cell 10
Aqp4 (astrocytes)	✓	✓	×	✓	✓	✓	✓	✓	✓	✓
Eomes (nIPCs, NB1)	×	×	×	✓	✓	✓	×	✓	×	×
Mxd3 (RG, nIPCs)	×	×	✓	×	✓	✓	✓	✓	✓	×
Nestin (RG, nIPCs, vasculature)	✓	✓	✓	✓	✓	✓	✓	✓	✓	✓
Wnt8b (RG, nIPCs)	×	×	×	×	×	×	✓	×	×	×
Calb2 (NB1, NB2, CR)	×	×	×	×	×	×	×	×	×	×
Cdk1 (nIPCs)	✓	✓	✓	×	✓	✓	✓	✓	✓	✓
Igfbp1 (nIPCs, NB1, NB2)	×	×	✓	×	×	×	×	✓	×	×

Supplementary Figure S2: Characterization of neurosphere cultures derived from hippocampi of newborn C57BL/6J x Cast/EiJ hybrid mice and their individual neural stem cells. **(A)** Images of neurospheres at day 3 of culture. Neurite-like extensions were recognizable occasionally (arrow). **(B)** Immunofluorescence image of *in vitro* differentiated neurospheres showing their capacity to generate neurons (Tuj1, green) and glial cells (GFAP, red). **(C)** Marker gene expression in a sample of single neurosphere cells identified using qRT-PCR (sc-GEM) to determine cell type characteristics. Marker genes were selected according to (Hochgerner et al., 2018) and represent the following cell types: RG = Radial glia-like, nIPC = Neural intermediate progenitor cells, NB1 = Neuroblast 1, NB2 = Neuroblast 2, CR = Cajal-Retzius cell. Genes: *Aquaporin-4 (Aqp4)*, *Eomesodermin (Eomes)*, *MAX Dimerization Protein 3 (Mxd3)*, *Nestin*, *Wnt Family Member 8B (Wnt 8b)*, *Calbindin 2 (Calb2)*, *Cyclin dependent kinase 1 (Cdk1)* and *Insulin Like Growth Factor Binding Protein 1 (Igfbp1)*.
