# Supplementary material for: Variable allelic expression of imprinted genes at the Peg13, Trappc9, Ago2 cluster in single neural cells
Source: Front Cell Dev Biol. 2022 Oct 12;10:1022422. doi: 10.3389/fcell.2022.1022422 (PMC9596773; doi:10.3389/fcell.2022.1022422)
Supplement: Supplementary file 4 [file DataSheet6.PDF]

Genomic feature	Reg - A	Reg - 2	Reg - B	Reg - C	Reg - 8	Reg - D	Reg - E
Position (Chr:15)	72,625,407-72,626,800	72,657,344-72,658,462	72,849,700-72,853,299	72,860,700-72,862,299	72,891,564-72,892,637	73,019,700-73,023,494	73,066,670-73,068,528
cHMM	En-P (4)	En-W (2)<br>En-P (3)	En-W (2)<br>En-S (2)<br>En-P (3 and 4)<br>En-Ws (3)	En-W (2)<br>En-P (3 and 4)	En-W (4)	En-W (2, 3 and 4)<br>En-P (3 and 4)<br>En-S (2, 3 and 4)	En-S (2 and 4) En-P (2, 3 and 4)
cCRE	enhD	enhD	enhD	enhD	enhD	enhD	enhD
ATAC	1,2,3,4	1,2,3,4	1,2,3,4	1,2,3,4	1,2,3,4	1,2,3,4	1,2,3,4
CTCF	X	2,5	2,5	X	X	1,2,3,4,5	2,3,4
POLR2A	1	5	5	X	5	5	1,5
DNAse HS	1,2,3,4	1,2,3,4	1,2,3,4	1,2,3,4	1,2,3,4	1,2,3,4	1,2,3,4
H3K4 me1	1,2,3,4,5	1,2,3,4,5	1,2,3,4,5	1,2,3,4,5	2,3,4,5	1,2,3,4,5	1,2,3,4,5
H3K4 me2	2,3,4	2,4	2,3,4	2,3,4	4	2,3,4	2,3,4
H3K4 me3	X	X	5	X	X	2,3,4,5	1,2,3,4,5
H3K27ac	2,3,5	2,3,4,5	1,2,3,4,5	2,3,4,5	2,3,4	1,2,3,4,5	1,2,3,4,5
H3K9ac	2,3,4	2,4	2,3,4	2,3,4	2,3	2,3,4	2,3,4

LEGEND		
CEREBELLUM = 1	FOREBRAIN = 2	MIDBRAIN = 3
HINDBRAIN = 4	OLFACTORY BULB = 5	FRONTAL CORTEX = 6

Supplementary Figure S6: Identification of potential brain-specific regulatory elements (Reg) across the *Peg13-Trappc9* locus. The candidate brain-specific regulatory regions were identified using the ENCODE3, Ensembl and UCSC Genome Browser databases to extract epigenetic and chromatin accessibility features (ChIP data for histone modifications, CTCF and RNA-polymerase II, DNAse I and ATAC hypersensitivity), which are associated with enhancer-like characteristics in the P0 mouse brain. Abbreviations correspond to ENCODE3 / UCSC Genome Browser annotations. cHMM = chromHMM (chromatin hidden Markov model). cCRE = candidate cis-regulatory element. Chromosome positions refer to the mouse GRCm38/mm10 genome version.
