# Supplementary material for: Variable allelic expression of imprinted genes at the Peg13, Trappc9, Ago2 cluster in single neural cells
Source: Front Cell Dev Biol. 2022 Oct 12;10:1022422. doi: 10.3389/fcell.2022.1022422 (PMC9596773; doi:10.3389/fcell.2022.1022422)
Supplement: Supplementary file 5 [file DataSheet3.PDF]

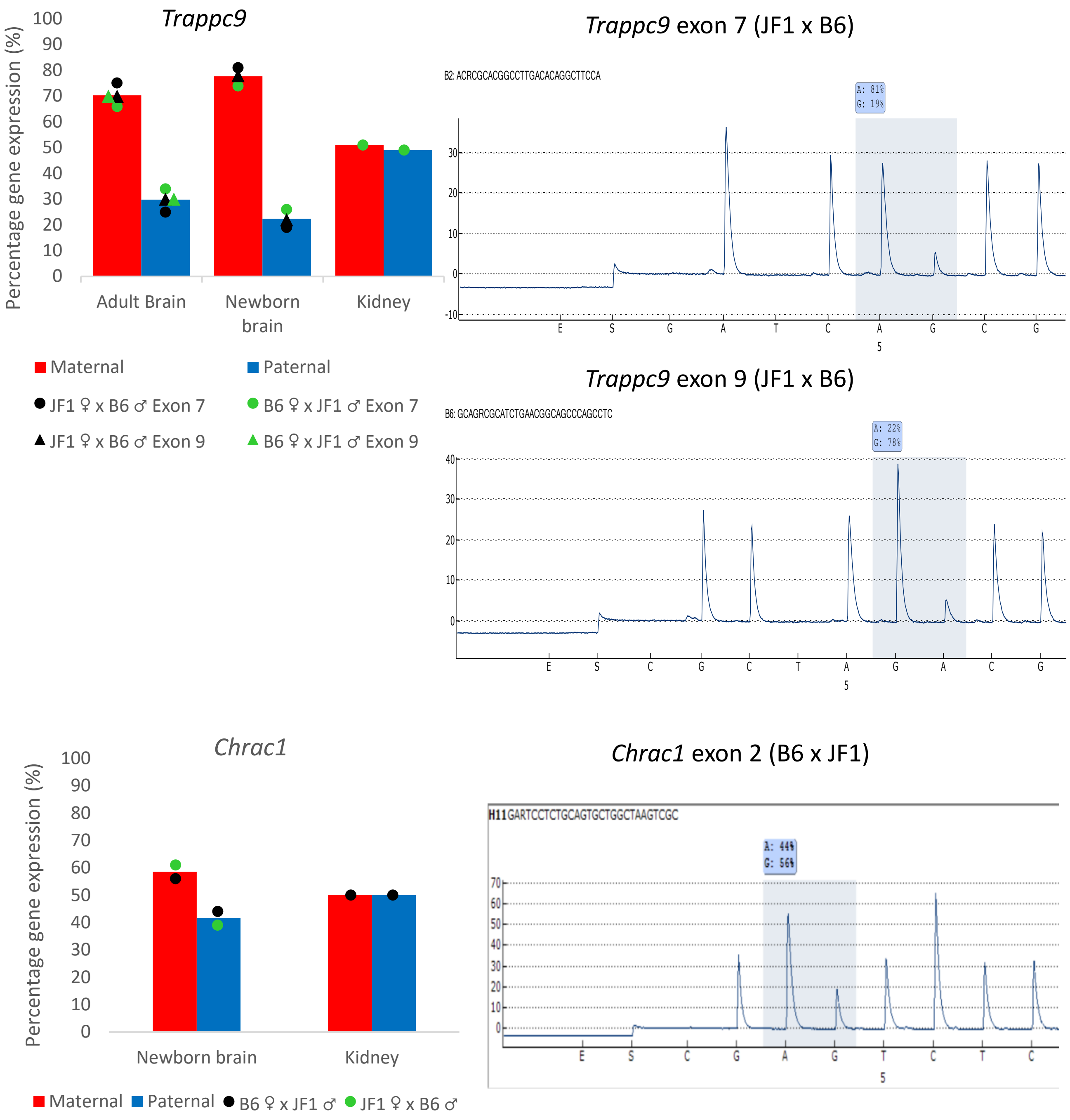

Supplementary Figure S3: Allelic expression of *Trappc9* and *Chrac1* in newborn and adult brain and adult kidney of C57BL/6J (B6) and *Mus musculus molossinus* (JF1) hybrid mice. Parental allelic expression was quantified via SNP pyrosequencing of cDNA. Average expression and example traces from newborn brain are shown. SNP IDs and exon locations are: *Trappc9*: rs31443481 located in exon 7 and rs31438077 located in exon 9; *Chrac1:* rs248258787 in exon 2. Points on the bar graphs represent individual pyrosequencing results from reciprocal crosses as shown in the legend.
