# Supplementary material for: Variable allelic expression of imprinted genes at the Peg13, Trappc9, Ago2 cluster in single neural cells
Source: Front Cell Dev Biol. 2022 Oct 12;10:1022422. doi: 10.3389/fcell.2022.1022422 (PMC9596773; doi:10.3389/fcell.2022.1022422)
Supplement: Supplementary file 6 [file DataSheet1.PDF]

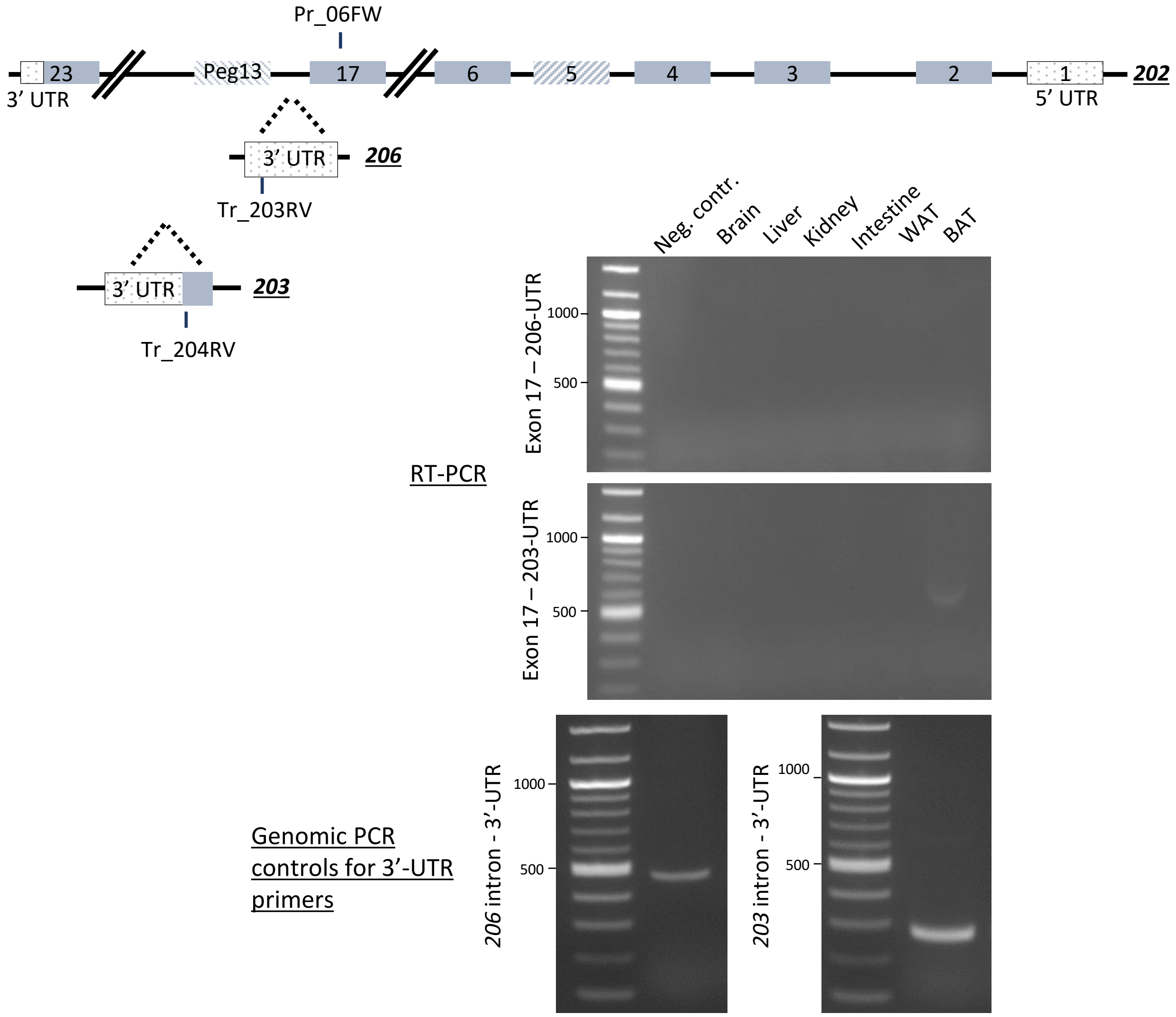

Supplementary Figure S1: Alternative, truncated transcript variants *206* and *203* of *Trappc9* could not be detected (for transcript scheme see Figure 1A). RT-PCR on total C57BL/6J RNA from multiple tissues using primer combinations specific for transcript *206* and *203* 3'-UTRs, respectively, combined with a shared upstream primer in exon 17 (indicated in the scheme with RV and FW annotations). Expected product sizes: 228 bp for exon 17 – *206*-UTR; 427 bp for exon 17 – *203*-UTR. No expression of these transcripts could be detected in multiple tissue samples, which were however positive for variant *202* (as shown in Figure 1). Positive controls of the 3'-UTR primers with respective intronic primers on genomic DNA confirmed their functionality in principle (expected products: 452 bp for *206;* 272 bp for *203*).
