# Supplementary material for: Variable allelic expression of imprinted genes at the Peg13, Trappc9, Ago2 cluster in single neural cells
Source: Front Cell Dev Biol. 2022 Oct 12;10:1022422. doi: 10.3389/fcell.2022.1022422 (PMC9596773; doi:10.3389/fcell.2022.1022422)
Supplement: Supplementary file 7 [file DataSheet5.PDF]

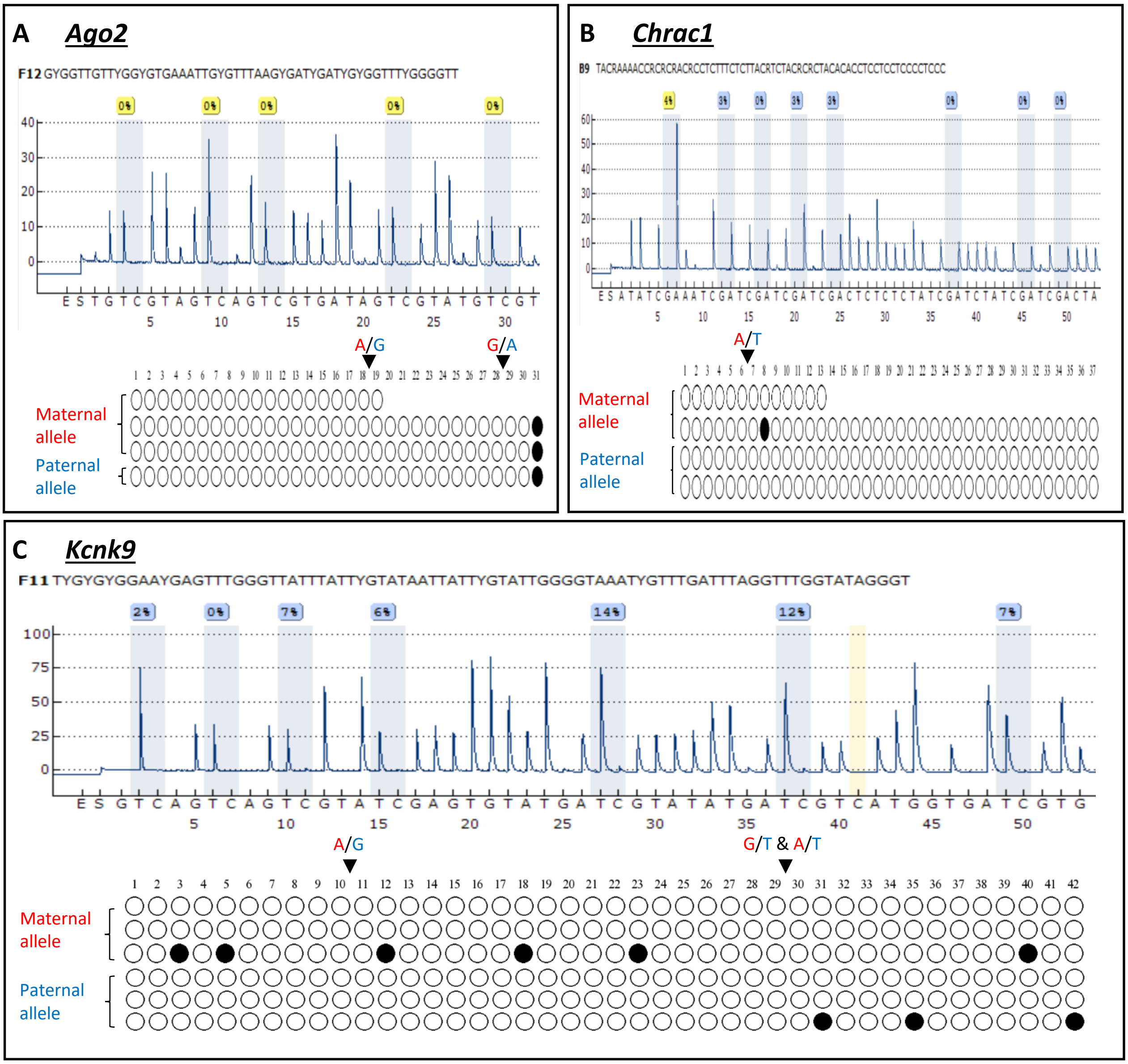

Supplementary Figure S5: Analysis of genomic DNA methylation at CGIs through bisulfite-treatment followed by pyrosequencing or Sanger sequencing of cloned samples. Data are for neural stem cells (hippocampal neurospheres) obtained from C57BL/6J x Cast/EiJ newborn F1 hybrids. **(A)** The promoter CGI at *Ago2* is unmethylated on both parental alleles in neural stem cells (pyrogram and Sanger sequencing; SNPs: RS257455001 and RS227718844). Black circle = methylated CpG; white circle = unmethylated CpG). **(B)** The promoter CGI at *Chrac1* is unmethylated on both parental alleles in neurospheres (pyrogram and Sanger sequencing; SNP: RS249421726). **(C)** The promoter CGI at *Kcnk9* is unmethylated on both parental alleles in neurospheres (pyrogram and Sanger sequencing; SNPs: RS583874465, RS219245628 and RS259286628).
