# Supplementary material for: Variable allelic expression of imprinted genes at the Peg13, Trappc9, Ago2 cluster in single neural cells
Source: Front Cell Dev Biol. 2022 Oct 12;10:1022422. doi: 10.3389/fcell.2022.1022422 (PMC9596773; doi:10.3389/fcell.2022.1022422)
Supplement: Supplementary file 8 [file DataSheet8.PDF]

Supplementary Table S2: List of single neural stem cells (NSCs) or differentiated neurons that showed unexpected equal bi-allelic, maternally biased bi-allelic or mono-allelic maternal expression of *Peg13* and their expression status of *Trappc9* and *Ago2* (related to Figures 3 - 5). There was no specific pattern of correlation between the allelic expression status of *Peg13* and the two other genes of interest. N/A = not analyzed.

Cell	*Peg13* expression	*Trappc9* expression	*Ago2* expression
**Expected expression**	**Mono-allelic paternal**	**Biased maternal**	**Biased maternal**
**Exp 1: Cell 1**	Biased maternal	Biased paternal	Biased paternal
**Exp 1: Cell 2**	Biased maternal	Biased paternal	equal biallelic
**Exp 1: Cell 3**	Biased maternal	Biased maternal	Biased paternal
**Exp 1: Cell 5**	Mono-allelic maternal	Biased paternal	Biased maternal
**Exp 2: Cell 9**	Biased maternal	N/A	N/A
**Exp 3: Cell 4**	Biased maternal	N/A	Biased maternal
**Exp 3: Cell 20**	Mono-allelic maternal	N/A	N/A
**Exp 6: Cell 1 (differentiated neuron)**	Biased maternal	Biased paternal	Biased maternal
**Exp 7: Cell 3**	Biased maternal	Mono-allelic paternal	Biased maternal
**Exp 7: Cell 8**	equal biallelic	Biased paternal	equal biallelic
**Exp 7: Cell 14**	Mono-allelic maternal	Biased maternal	Mono-allelic paternal
**Exp 7: Cell 17**	Biased maternal	Mono-allelic maternal	Biased maternal
**Exp 7: Cell 4**	Biased maternal	Biased paternal	Biased paternal
