# Supplementary material for: Variable allelic expression of imprinted genes at the Peg13, Trappc9, Ago2 cluster in single neural cells
Source: Front Cell Dev Biol. 2022 Oct 12;10:1022422. doi: 10.3389/fcell.2022.1022422 (PMC9596773; doi:10.3389/fcell.2022.1022422)
Supplement: Supplementary file 3 [file DataSheet4.PDF]

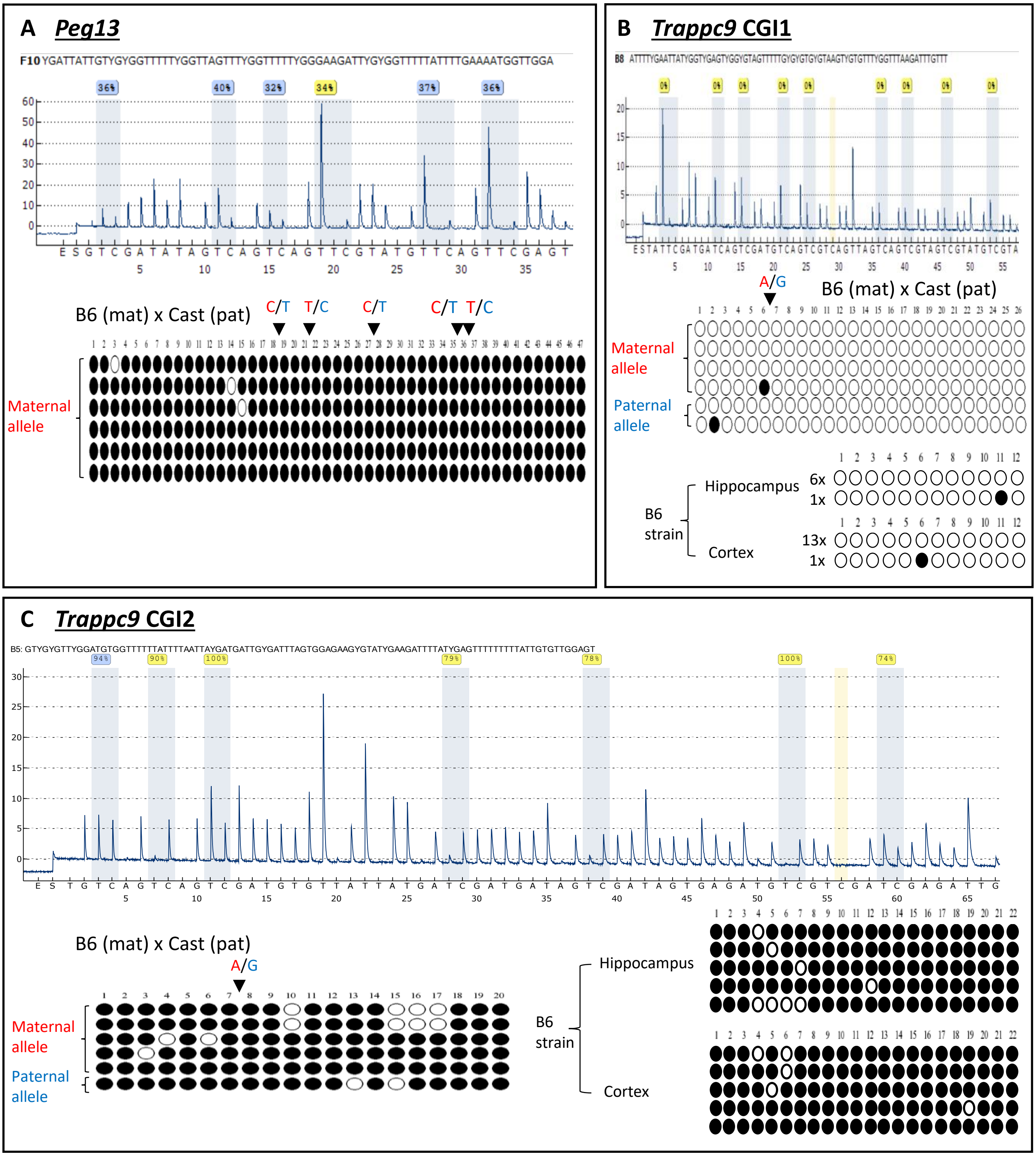

Supplementary Figure S4: Analysis of genomic DNA methylation at CGIs through bisulfite-treatment followed by pyrosequencing or Sanger sequencing of cloned samples. Data are for neural stem cells (hippocampal neurospheres) obtained from C57BL/6J x Cast/EiJ newborn F1 hybrids, unless otherwise indicated. **(A)** The *Peg13* differentially methylated region (DMR) shows an average methylation of ~36% at indicated CpGs (pyrogram), close to the expected 50%. Sanger sequencing confirms presence of methylation on the maternal allele (black circle = methylated CpG; white circle = unmethylated CpG). Allelic SNPs between the numbered CpGs are RS242845136, RS31423568, RS31423567, RS238259968 and RS31423566. **(B)** The *Trappc9* promoter/exon 1 CGI is unmethylated on both parental alleles in neurospheres (Sanger sequencing; SNP: RS31441779 between the 6-7th CpG), C57BL/6J adult hippocampus and cortex tissue (Sanger sequencing) and in newborn F1 hybrid kidney (pyrogram). **(C)** The *Trappc9* exon 2 CGI is fully methylated on both parental alleles in neurospheres (pyrogram and Sanger sequencing; SNP: RS31440851 between the 7-8th CpG), as well as in adult hippocampus and cortex (C57BL/6J; additional Sanger sequencing).
